# Synergistic Evolution Law of Surface Work Hardening and Contact Fatigue in Wind Power Bearing Steel Pretreated by Ultrasonic Impact

**DOI:** 10.3390/ma19194143

**Published:** 2026-09-28

**Authors:** Peiding Liu, Fengyang Fu, Liangyu Wang, Jiacai Han, Yihan Jiang, Zhilong Xu, Peihan Lin, Fei Sun, Tong Ran

**Affiliations:** 1 Sichuan Yanyuan Huadian New Energy Co., Ltd., Liangshan 615799, China; myloxylotolpd@163.com (P.L.); qzhan745@gmail.com (F.F.); m18009043752@163.com (L.W.); hanjiacai2026@163.com (J.H.); 15883780721@163.com (Y.J.); 2 College of Marine Equipment and Mechanical Engineering, Jimei University, Xiamen 361021, China; zhilong.xu@163.com (Z.X.); 202412855017@jmu.edu.cn (P.L.); sunfei0703@163.com (F.S.)

**Keywords:** ultrasonic impact treatment (UIT), 100CrMnMoSi8-4-6 bearing steel, rolling contact fatigue, secondary work hardening

## Abstract

**Highlights:**

- Ultrasonic impact can achieve surface finishing and surface gradient strengthening of wind power bearing steel in a single step, demonstrating engineering value as a *potential substitute for oilstone final finishing*.
- High static load simultaneously optimizes surface integrity, residual stress fields, and multi-scale microstructure; quantitative EBSD analysis elucidates the synergistic strengthening mechanisms of grain refinement and carbide dispersion.
- Within the investigated static load range (500–1000 N), the secondary hardening increment decreases as the static pressure increases.
- A new process evaluation criterion is established that balances static surface properties and service dynamic hardening behavior, providing a theoretical basis for life-extension manufacturing of wind power bearing rollers.

**Abstract:**

To address rolling contact fatigue failure in 100CrMnMoSi8-4-6 wind power bearing rollers and the limitation of conventional oilstone finishing, this study applies ultrasonic impact treatment (UIT) at two static loads (500 N and 1000 N), systematically investigating effects on surface integrity. Under laboratory rolling contact conditions corresponding to the typical contact stresses encountered in actual wind turbine bearings, gradient microstructure, residual stress, hardness, secondary work hardening, and fatigue life. Results show that UIT simultaneously achieves surface smoothing and gradient strengthening; under 1000 N, roughness drops from 0.41 to 0.11 μm, residual stress reaches −842 MPa, hardness approaches 900 HV_0.5_, and grain size refines to 1.44 μm with increased low-angle boundaries and fragmented carbides. After rolling contact, the secondary hardening increments for the as-received specimen, the 500 N specimen, and the 1000 N specimen were 4.98%, 3.63%, and 2.11%, respectively; this indicates that the subsequent contact-induced hardening response is closely related to the initial strengthening state established by the UIT process. The 500 N specimen exhibited a significant secondary hardening capability despite having achieved initial strengthening, whereas the 1000 N specimen, although possessing a lower secondary hardening increment, achieved the highest rolling contact fatigue life under the experimental conditions of this study owing to its higher initial hardness, residual compressive stress, and more pronounced microstructural refinement. The fatigue life of the specimens treated with 500 N and 1000 N was increased by 378% and 1214%, respectively, compared to the as-received specimen. These results demonstrate that, within the range of parameters investigated, there exists a static load-dependent relationship between the initial UIT strengthening and the subsequent cyclic contact hardening; this suggests that when evaluating UIT parameters, both the initial surface condition and its subsequent evolution under rolling contact should be taken into account simultaneously. Therefore, the UIT technique holds potential as a comprehensive surface finishing and strengthening treatment method for 100CrMnMoSi8-4-6 bearing rollers; however, its optimal processing range and transferability to actual bearing raceways require further validation.

## 1. Introduction

Rolling bearings, as core fundamental components in modern mechanical transmission systems, are widely used in critical fields such as automobiles, trains, aircraft, and energy equipment [1,2]. With the continuous advancement of modern industry toward high speed, heavy load, and extreme environmental adaptability, the service conditions of bearings are becoming increasingly demanding. Particularly in the field of wind power generation, mainshaft bearings are subjected not only to enormous radial and axial loads but also to complex alternating stresses and impact loads induced by wind speed fluctuations [3]. Under such extreme conditions, the contact fatigue performance of bearing materials directly determines the reliability and maintenance costs of the power unit. 100CrMnMoSi8-4-6, as a high-performance high-carbon chromium bearing steel [4], has become the preferred material for mainshaft bearing rollers in large wind turbines owing to its excellent hardenability, high strength, and good wear resistance. Nevertheless, such bearings still face severe challenges from rolling contact fatigue during long-term service [5].

Rolling contact fatigue is one of the primary failure modes of bearings. For wind turbine bearing rollers, the high-intensity rolling contact imposes extremely stringent requirements on surface roughness [5,6]. Microscopic stress concentrations caused by surface roughness significantly accelerate the initiation of fatigue cracks [7,8], making the finishing process crucial. Oilstone, as the traditional final finishing method, can effectively reduce the surface roughness of rollers and extend fatigue life to a certain extent. However, under modern high-cleanliness lubrication and high-surface-quality manufacturing processes, relying solely on roughness reduction is no longer sufficient to meet the ever-increasing demands for fatigue life. Under repeated Hertzian contact stresses, the maximum orthogonal shear stress is distributed at a certain depth beneath the material surface [9]. Once a crack initiates on the surface, it propagates along a direction at a certain angle to the surface, eventually leading to material spalling and complete loss of bearing function [10]. Therefore, how to optimize the stress state and microstructure of the material surface layer through surface modification techniques to suppress crack initiation and propagation is a key scientific issue for enhancing the fatigue life of wind power bearing rollers.

To address the above challenges, researchers have proposed various surface strengthening techniques [11,12,13]. Pape et al. [14,15] investigated the effects of mechanical surface treatments, including hard turning and deep rolling, on the surface integrity of rolling bearings. Their results showed that these processes could effectively reduce surface roughness and introduce beneficial compressive residual stresses in the surface and subsurface regions, thereby improving rolling contact fatigue performance and increasing the fatigue life of roller bearings by approximately 2.5 times. The authors further highlighted the importance of the combined effects of surface topography, work hardening, and residual stress state on rolling contact fatigue performance, providing an important basis for improving bearing service performance through mechanical surface strengthening. Dong et al. [10] incorporated rare earth elements combined with mechanical rolling to fabricate a 700 μm nanocrystalline gradient strengthening layer, effectively improving contact fatigue performance. Xia et al. [7] found that surface roughness plays a critical role in contact fatigue life, with RCF life extending as surface roughness decreases. Steven et al. [16] observed that high surface stresses induce localized plastic deformation and micropitting, all of which exacerbate contact fatigue. Cerullo et al. [17] investigated the very-high-cycle fatigue behavior of wind-turbine roller bearings using a two-dimensional plane-strain finite element model, in combination with Hertzian contact theory, elastohydrodynamic lubrication theory, and the Dang Van multiaxial fatigue criterion. Their results indicated that fatigue failure tends to initiate in the subsurface region subjected to high cyclic stresses and that the hardness and its depth distribution have a significant influence on the fatigue safety of bearings, highlighting the importance of both the subsurface stress state and gradient mechanical properties in the assessment of rolling contact fatigue. Wu et al. [18] investigated the surface integrity and contact fatigue performance of 18CrNiMo7-6 steel after ultrasonic impact treatment, finding that the crack initiation region shifted from the surface to the subsurface and that crack type transitioned from tensile to shear mode. Zhu et al. [19] studied the effect of USRP on G20Cr2Ni4A steel, demonstrating that ultrasonic impact not only enhanced the resistance of the internal microstructure to crack initiation and propagation but also deepened the crack initiation site within the internal microstructure, thereby prolonging the RCF life of specimens. Although ultrasonic impact technology has attracted considerable attention for its ability to significantly improve metallic surface integrity [20,21], it does more than simply reduce surface roughness to levels comparable to or even better than oilstone finishing through high-frequency mechanical impact-induced severe elastoplastic deformation in the surface layer [22]. More importantly, it introduces a deep compressive residual stress field in the surface layer [23]. Such compressive residual stresses can effectively counteract service-induced tensile stresses and delay crack initiation and propagation [24,25]. Ultrasonic impact thus achieves a win–win strengthening effect by meeting the stringent surface roughness requirements while simultaneously introducing beneficial compressive residual stresses against rolling contact fatigue [18,26,27]. As discovered by Qu et al., ultrasonic surface rolling treatment significantly improves the rolling contact fatigue life of 25CrNi2MoV shaft steel at high lubrication viscosities by refining the grain structure, increasing hardness, introducing compressive residual stresses, and reducing surface roughness.

Although previous studies have demonstrated the positive effects of ultrasonic rolling in combating contact fatigue [28,29], research on 100CrMnMoSi8-4-6 bearing steel, particularly regarding rolling contact fatigue behavior under simulated real wind turbine operating conditions, remains relatively scarce. In fact, ultrasonic rolling not only introduces surface compressive residual stresses and a nanocrystalline gradient structure to enhance fatigue resistance but also significantly reduces surface roughness. UIT has the potential to simplify or partially replace conventional oilstone finishing in applications requiring both surface finishing and surface strengthening. By adopting ultrasonic rolling, the stringent roughness requirements of rollers can be met while simultaneously achieving strengthening effects, thus integrating “friction reduction” and “fatigue resistance” into the actual manufacturing chain of rollers. In view of this, the present study aims to investigate the enhancement mechanism of ultrasonic impact treatment on the rolling contact fatigue life of 100CrMnMoSi8-4-6 bearing rollers. Under laboratory rolling contact conditions corresponding to the typical contact stresses encountered in actual wind turbine bearings, by comparing the fatigue lives of untreated and ultrasonic-impact-treated specimens under simulated wind turbine operating conditions, combined with microstructural characterization and residual stress measurements, this study provides an in-depth analysis of the inhibitory effects of the surface nanocrystallized structure and compressive residual stress field introduced by ultrasonic impact on subsurface crack initiation and propagation. This study establishes a correlation mechanism between ultrasonic static load and rolling contact secondary hardening, demonstrating that higher static load does not necessarily yield better comprehensive service performance, and identifies an optimal static pressure range that balances initial surface strength and service hardening potential. This provides a new criterion for the trade-off optimization of ultrasonic impact process parameters for wind power bearing rollers, addressing the limitation of existing studies that rely solely on static mechanical properties as process evaluation indicators.

## 2. Materials and Methods

### 2.1. Experimental Materials

In this study, the base material selected for the contact fatigue tests and ultrasonic impact treatment is a high-hardenability bearing steel, 100CrMnMoSi8-4-6 (Luoyang Bearing Science & Technology Co., Ltd., Luoyang, China). To achieve an excellent combination of strength and toughness as well as superior fatigue resistance, the roller specimens were subjected to an isothermal quenching process to form a lower martensite structure. After heat treatment, the surface hardness of the specimens was strictly controlled within the range of 59–63 HRC, while the core hardness was maintained at ≥55 HRC, ensuring sufficient load-bearing capacity when the material is subjected to high Hertzian contact stress. Meanwhile, to minimize dimensional instability caused by the decomposition of retained austenite during service, the surface-retained austenite content was strictly controlled to ≤5%. Apart from the above key indicators, all other heat treatment process parameters and quality control procedures strictly comply with the GB/T 34891 standard. The specific chemical composition analysis results of this material are presented in Table 1. The untreated initial specimens were finished by conventional oilstone polishing and were used as the baseline control for evaluating the surface finishing and strengthening effects of UIT. Under the experimental conditions investigated in this study, the above results demonstrate that UIT has potential as an integrated surface finishing and strengthening process for 100CrMnMoSi8-4-6 bearing rollers. However, its applicability to inner and outer raceways and complete bearing systems requires further component-specific experimental validation.

To meet the clamping requirements of the contact fatigue testing machine and the ultrasonic impact treatment equipment, the heat-treated rollers were first fixed on a wire electrical discharge machining (wire-EDM) platform and machined into specific geometric dimensions according to the experimental design (as shown in Figure 1). After wire-EDM, all specimen surfaces were subjected to systematic mechanical grinding and precision polishing to eliminate the cutting-affected layer and reduce the influence of initial surface defects on fatigue life. The final surface roughness was verified using a profilometer to ensure Ra ≤ 0.8 μm, thereby satisfying the stringent surface integrity requirements for contact mechanics experiments. The prepared standard specimens were then used for subsequent ultrasonic impact treatment and comparative fatigue tests.

### 2.2. Experimental Setup

The experimental setup for the ultrasonic impact treatment employed in this study is illustrated in the figure. The system primarily consists of an ultrasonic generator, a transducer, a horn, and an impact actuator. During the experiments, the system operated at a resonant frequency of *f* = 27 ± 0.8 kHz, which is an intrinsic physical parameter of the equipment and was kept constant throughout the experiments to ensure the stability of energy transmission. The vibration amplitude was precisely adjusted via the control cabinet to meet the requirements of different experimental conditions.

To ensure the uniformity of the impact effect and wear resistance, a tungsten carbide ball head with a diameter of 14 mm was selected as the impact tool head. Regarding the process parameter settings, the static load was applied through a spring-loading system and was monitored in real time with feedback control via the display. During the experiments, the feed per revolution of the machine tool was uniformly set to 1 mm/rev, the feed rate to 100 mm/min, and the spindle speed to 100 r/min. The feed per revolution, feed rate, and spindle speed were all treated as fixed quantitative control parameters and were kept constant throughout the specimen preparation process. Only the amplitude and static load were varied as control variables to accurately assess their effects on the surface integrity of the material.

The contact fatigue tests in this study were conducted on an RCTF-6000 rolling contact fatigue testing machine, as shown in Figure 2. To clarify the correspondence between the laboratory rolling contact fatigue (RCF) test conditions and the representative rolling-contact loading conditions of wind-turbine bearings, the principal test parameters were explicitly defined. The specimen geometry is shown in Figure 1b. The rotational speed was set to 10,000 r/min, corresponding to a loading frequency of 166 Hz. During the test, Tribol GR SW 460-1 lubricant was supplied to the rolling contact region by drip lubrication at a flow rate of 3 L/min. A contact load of 324 N was applied, corresponding to a maximum Hertzian contact stress of 2.4 GPa. This contact-stress level was selected with reference to representative contact stresses encountered under operating conditions of wind-turbine bearings, thereby establishing correspondence between the laboratory test and practical bearing applications in terms of this key loading parameter. It should be noted, however, that the present test was conducted under controlled laboratory conditions and was intended primarily to comparatively evaluate the RCF performance of specimens under different treatment conditions, rather than to fully reproduce the complex service conditions of an actual wind-turbine bearing. The testing machine was operated under a nominal pure-rolling configuration, in which the specimen was actively driven while the idler rollers rotated passively through contact with the specimen; therefore, no slide-to-roll ratio was intentionally imposed (nominal SRR = 0). Nevertheless, small amounts of microslip or spin may occur during actual bearing operation, and their effects on surface hardening and fatigue damage require further investigation. Accordingly, the conclusions of the present study are primarily applicable to rolling-dominated contact conditions.

According to Hertzian contact theory and its underlying assumptions, an elliptical contact area is formed between the idler roller and the equivalent component, with major and minor axes of 2a and 2b, respectively. The corresponding maximum Hertzian contact pressure, *P*_0_ was calculated using Equation (1); this equation is automatically provided by the equipment.

(1) $$P_{0}=\frac{3P}{2\pi ab}$$

The basic working principle and internal structure of the machine are illustrated in Figure 2c. The specimen is clamped on an air spindle and positioned between two pressing idler rollers. The interaction of the two idler rollers ensures that the specimen is not subjected to additional bending loads, and the specimen drives the two pressing idler rollers in pure rolling motion. A cylinder drives the pressing idler rollers via a swing arm to apply a contact load to the specimen; the applied load equals the thrust force of the cylinder and is measured by a pressure sensor installed on the cylinder. A vibration sensor is mounted on the swing arm. When contact fatigue damage, such as spalling pits, occurs on the specimen, the system vibration increases significantly. Once the vibration amplitude reaches a preset threshold (set to 6 μm in this study based on previous experimental experience), the test stops automatically. With respect to the contact fatigue test parameters, only the applied load was considered as the single variable, while the spindle speed was fixed at 10,000 r/min as a constant. During the rolling contact fatigue test, the vibration amplitude of the system gradually increased with the initiation and propagation of fatigue cracks and the development of spalling damage. The test was automatically terminated when the vibration amplitude reached 6 μm. This threshold was not used merely as an equipment-control parameter but as a termination criterion corresponding to the occurrence of identifiable fatigue damage. After the fatigue tests, clear fatigue-spalling damage associated with reaching the 6 μm vibration threshold was observed for specimens under all treatment conditions. Therefore, a vibration amplitude of 6 μm was adopted as a unified fatigue failure criterion to ensure the comparability of fatigue-life results among different specimens. It should be emphasized that the “fatigue life” reported in this study is defined as the number of rolling-contact cycles required for the vibration amplitude to reach the 6 μm threshold, rather than the number of cycles corresponding to complete loss of load-carrying capacity or extensive macroscopic spalling.

To ensure the reliability of the fatigue-life evaluation under different treatment conditions, three independent specimens were tested for each condition. Each specimen was independently subjected to one rolling contact fatigue test, and the number of cycles corresponding to the predefined fatigue failure criterion was recorded. The fatigue-life results for each condition are reported as mean ± standard deviation. In addition, a Weibull distribution analysis was performed using the individual fatigue lives of the three independent specimens to further characterize the fatigue-life distribution and scatter under different treatment conditions.

### 2.3. Mechanical Properties and Microstructure Testing

The microhardness distribution of the specimens was measured using a FALCON 511 fully automatic Vickers hardness tester (INNOVATEST B.V., Maastricht, The Netherlands). The test load was set to 0.1 kgf (≈0.98 N) with a dwell time of 10 s. To comprehensively evaluate the gradient structural strengthening effect introduced by the ultrasonic impact treatment in the near-surface region, the hardness measurement path was oriented perpendicularly from the specimen surface toward the core along the cross-section. Point-by-point measurements were conducted using a gradient spacing method to obtain an accurate hardness–depth profile. Hardness measurements were performed in strict accordance with GB/T 230.1-2018 [30]. The center-to-center distance between adjacent indentations was set to ≥50 μm, while the corresponding indentation diagonal length was approximately 15–20 μm, giving a spacing-to-diagonal ratio of approximately 2.5–3.3. This spacing satisfies the standard requirement for sufficient separation between adjacent indentations and effectively minimizes interference between the work-hardened/deformed zones generated by neighboring indentations.

Residual stress analysis was conducted using an LXRD X-ray stress analyzer manufactured by Proto (Proto Manufacturing Ltd. is located in Oldcastle, ON, Canada). Cr-Kα radiation was selected as the X-ray source, and the sin^2^ψ method was employed for stress calculation. To reveal the distribution of residual compressive stress beneath the subsurface, the specimen surface was progressively removed layer by layer via electrolytic polishing (parameters: 30 V for 10 s), and gradient residual stress measurements were subsequently performed. Through this method, particular attention was paid to the maximum peak value of the residual compressive stress and its corresponding depth position, thereby quantitatively evaluating the strengthening depth and the degree of strengthening induced by the ultrasonic impact treatment on the near-surface layer. To ensure the reliability and repeatability of the experimental data, at least three independent replicate experiments were carried out for each set of process parameters, and the arithmetic mean was ultimately used as the analytical data in this study.

To characterize the depth-dependent distribution of residual stresses in the surface and subsurface regions after ultrasonic impact treatment (UIT), X-ray diffraction (XRD) residual-stress measurements were performed. A Proto LXRD X-ray residual-stress analyzer was used with a Cr target and Cr-Kα characteristic radiation. Residual stresses were measured and calculated using a side-inclination geometry based on the sin^2^ψ method, with an X-ray diffraction scanning range of 146–156°.

To obtain the residual-stress distribution at different depths, the specimen surface was progressively removed by successive electropolishing. After each electropolishing step, the residual stress on the newly exposed surface was measured by XRD, and the cumulative material-removal depth relative to the original surface was used as the depth coordinate. By repeatedly performing the sequence of “electropolishing–residual-stress measurement,” residual-stress values at different depths were obtained and used to construct the residual-stress gradient along the depth direction. It should be noted that progressive layer removal during electropolishing may alter the original mechanical equilibrium of the specimen and may induce a certain degree of elastic stress relaxation and redistribution. No additional correction for layer-removal-induced stress relaxation or redistribution was applied in the present study. Therefore, the measured residual-stress depth profiles are mainly used to characterize and compare the relative variation in residual-stress gradients under different UIT conditions, while the absolute stress values at larger material-removal depths should be interpreted cautiously in view of the possible influence of successive layer removal.

To systematically evaluate the effects of ultrasonic impact treatment (UIT) on the surface integrity and microstructural evolution of the material, a VK-X1000 laser confocal microscope (KEYENCE CORPORATION, Osaka, Japan) was first employed to perform three-dimensional topography scanning of the specimen surfaces, allowing for precise determination of the initial surface state and the variation in surface roughness after UIT.

Surface roughness measurements were performed according to ISO 4288 and GB/T 6062 [31]. The cutoff wavelength was set to λc = 0.08, and an evaluation length of 700 μm, exceeding 5λc, was used. The measurement direction was along the axial direction of the specimen (along the generatrix and perpendicular to the machining texture). One profile curve was extracted from each measurement trace, and the profile exhibiting the highest roughness value (*Ra*) was selected as the representative result for surface roughness characterization.

Subsequently, the specimens were chemically etched with a nital solution to clearly reveal their metallographic structural features. For further in-depth investigation into the surface-layer grain refinement mechanism and grain orientation evolution induced by UIT, electron backscatter diffraction (EBSD) analysis was conducted using a Carl Zeiss Crossbeam 550 field emission scanning electron microscope (FE-SEM, Carl Zeiss Oberkochen, Oberkochen, Germany). During the measurements, an accelerating voltage of 20 kV and a probe current of 100 nA were applied to obtain high-resolution diffraction patterns; step size selection, 0.12 μm. In the data acquisition and analysis stage, phases including body-centered cubic (BCC), face-centered cubic (FCC), and cementite (Fe_3_C) were simultaneously identified and indexed, enabling accurate interpretation of the grain refinement behavior in the multiphase microstructure. The same instrument was also used to observe the macroscopic and microscopic morphologies of the specimen surfaces after the contact fatigue tests, with particular attention to capturing surface spalling pits and the propagation paths of fatigue cracks. The collected data are post-processed using AZtecCrystal software (2026 version), with an automated cleaning routine employed to remove anomalous data points such as stray peaks; additionally, a criterion based on five adjacent points is applied to eliminate zero-resolution points, thereby improving the quality of the EBSD data and the reliability of the KAM analysis.

## 3. Results

### 3.1. Mechanical Properties Testing

#### 3.1.1. Surface Roughness

Figure 3 illustrates the improvement in surface roughness after UIT. In the initial state, the specimen surface exhibited a roughness Ra of 0.41 μm, with distinct and unevenly distributed machining marks that were clearly visible. After UIT with a static load of 500 N and an amplitude of 10 μm, the surface roughness was significantly reduced to 0.13 μm, the machining marks largely disappeared, and the surface became flatter and denser. When the impact load was further increased to 1000 N, the surface roughness decreased to 0.11 μm; although the reduction tended to level off, the surface finish and uniformity continued to improve. The experimental results indicate that ultrasonic impact treatment can effectively eliminate the original machining marks, significantly reduce surface roughness, and achieve a pronounced surface strengthening and leveling effect.

The untreated initial specimens were finished by conventional oilstone polishing and were used as the baseline control for evaluating the surface finishing and strengthening effects of UIT.

The experimental results demonstrate that, under a constant amplitude, ultrasonic impact treatment (UIT) reduced the surface roughness from the initial Ra of 0.41 μm to 0.13 μm under a static load of 500 N, and further down to 0.11 μm under 1000 N. This continuous improvement is primarily attributed to the increase in static load. The enhanced static load significantly increases the Hertzian contact stress between the impact pin and the workpiece surface, inducing more severe plastic deformation in the surface layer. Consequently, the micro-asperity peaks corresponding to the initial machining marks are crushed and directionally filled into the adjacent micro-valleys, effectively suppressing the undulations of the micro-topography. Simultaneously, a higher static load enhances the coupling and transmission efficiency of the ultrasonic vibration energy into the surface layer, increasing the accumulated equivalent plastic strain within the impact-affected zone. This allows for the asperity peaks to be thoroughly flattened and the micro-grooves left by the original machining marks to be further closed or eliminated. Moreover, the increase in static load facilitates a more uniform impact path coverage with a high overlap rate, thereby improving the uniformity of the surface integrity and driving the surface toward an ultra-low roughness state. In summary, the increase in static load serves as the dominant mechanical factor responsible for the further reduction in roughness from 0.13 μm to 0.11 μm and the elimination of the machining mark morphology, fully confirming the critical role of static load in optimizing the surface micro-geometry during the UIT process.

Notably, no obvious large-scale periodic surface waviness induced by UIT was observed in either the three-dimensional surface topographies or the representative profiles. The dominant surface features remaining after treatment were primarily associated with the original machining traces, whose characteristic scale was substantially larger than the local microscopic undulations induced by ultrasonic impacts. Therefore, under the material and processing conditions investigated in this study, and consistent with our previous study [32], the primary effect of UIT was to reduce the height of microscopic peaks and valleys and homogenize the pre-existing surface texture, rather than to introduce pronounced large-scale geometric irregularities.

#### 3.1.2. Residual Stress

As shown in Figure 4, the residual stress distribution along the depth direction of the specimens exhibits a typical gradient characteristic. The surface of the untreated base specimen presents only a low magnitude of initial residual stress, which mainly originates from the mechanical extrusion and thermoplastic deformation between the cutting tool and the material during the turning process. Owing to the localized plastic flow occurring in the surface layer during turning, followed by stress redistribution upon cooling and elastic recovery, a relatively small residual stress is generated in the surface region. However, its affected depth is limited, and the overall level of residual compressive stress remains low.

It should be noted that the above residual-stress depth profiles were obtained by combining successive electropolishing with X-ray residual-stress measurements. Since progressive material removal may induce elastic relaxation and redistribution of the original residual-stress field, no additional correction for this effect was applied in the present study. Therefore, the residual-stress peak observed at approximately 200 μm should be interpreted as the apparent maximum subsurface compressive residual stress measured under the present experimental procedure, rather than as the undisturbed residual-stress peak originally existing at the corresponding depth in the intact specimen. Accordingly, these measurements are primarily used to compare the relative changes and depth-gradient characteristics of residual stress under different static-load conditions.

In contrast, significant residual compressive stresses were introduced into the surface layer of all specimens subjected to ultrasonic impact treatment, and these stresses gradually decayed toward zero with increasing depth. Under both the 500 N–10 μm and 1000 N–10 μm conditions, the peak residual compressive stress appeared at a depth of approximately 200 μm from the surface, after which it progressively relaxed toward the core. This phenomenon is consistent with the Hertzian contact theory and the plastic deformation mechanism of ultrasonic impact treatment, whereby the maximum shear stress occurs not at the very surface but in the subsurface region [32]. Consequently, the peak residual compressive stress typically lags behind the surface position.

The two fields exhibit substantial overlap in the subsurface region: the compressive residual stress mainly extends over approximately 0–150 μm, while the critical subsurface shear-stress region is mainly distributed within approximately 0–200 μm [32]. This spatial overlap suggests that the UIT-induced compressive residual-stress field can beneficially modify the local cyclic stress state within the mechanically critical subsurface region, thereby reducing the effective fatigue-driving stress and potentially suppressing subsurface fatigue-crack initiation and propagation. This interpretation is consistent with the substantially improved rolling contact fatigue performance observed for the 1000 N specimen, which exhibited an approximately 1214% increase in fatigue life relative to the initial specimen.

Furthermore, as the static load increased from 500 N to 1000 N, the residual compressive stress levels in both the surface layer and the subsurface were further enhanced. The maximum surface value increased from −691 MPa to −734 MPa, while the subsurface maximum increased from −822 MPa to −842 MPa. The depth of the compressive stress-affected layer also increased significantly, by approximately 50 μm. This is primarily because, under a higher static load, the contact stress between the impact pin and the material increases, allowing for more impact energy to be transmitted into the material interior, thereby inducing more severe plastic deformation and dislocation accumulation. Meanwhile, the high-frequency cyclic impacts suppress the elastic recovery of the surface layer, causing the plastic strain to accumulate continuously and ultimately forming a more stable and deeper residual compressive stress field.

#### 3.1.3. Hardness

As shown in Figure 5, the surface microhardness of the 100CrMnMoSi8-4-6 bearing steel was significantly improved after ultrasonic impact treatment and exhibited a pronounced gradient distribution. The average microhardness of the initial specimen was 750.7 ± 46.3 HV_0.5_, while under the 500 N–10 μm and 1000 N–10 μm UIT parameters, the average hardness increased to 781.5 ± 31.1 HV_0.5_ and 803.9 ± 42.2 HV_0.5_, respectively. The 500 N–10 μm specimen exhibited an approximately 4.1% increase compared to the initial state, while the 1000 N–10 μm specimen showed an increase of approximately 7.1%. As can be seen from Figure 5a, the hardness of the initial specimen fluctuated only slightly over the entire measured depth range, remaining within 730–780 HV_0.5_. In contrast, after UIT, the hardness in the near-surface region increased significantly. For the 1000 N–10 μm specimen, the hardness within the 0–100 μm depth range reached a maximum of approximately 900 HV_0.5_, representing an increase of about 18–20% relative to the initial specimen; the surface hardness of the 500 N–10 μm specimen was approximately 850–860 HV_0.5_, corresponding to an increase of about 13–15%. With increasing depth, the hardness gradually decreased, began to stabilize at a depth of approximately 300–500 μm, and eventually approached the core hardness level. This indicates that UIT produced a distinct gradient-strengthened layer with an effective strengthening depth of approximately 400 μm. Compared with the 500 N–10 μm parameter, the 1000 N–10 μm specimen exhibited a higher hardness level and a deeper strengthened layer. Within the 0–250 μm range, the hardness of the 1000 N–10 μm specimen was overall 20–40 HV_0.5_ higher than that of the 500 N–10 μm specimen. This is because the higher static load increases the contact stress and impact energy, leading to greater accumulation of plastic strain in the surface layer and the introduction of a higher level of residual compressive stress. The residual compressive stress not only suppresses dislocation recovery but also retards micro-crack initiation, thereby further enhancing the surface hardening effect. Moreover, the higher impact energy promoted a greater degree of microstructural refinement in the surface layer, extending the thickness of the strengthened layer from approximately 300 μm under the 500 N condition to close to 500 μm.

Regarding the hardness scatter, the standard deviation of the initial specimen reached ±46.3 HV_0.5_, indicating a certain degree of inhomogeneity in the original microstructure. After UIT at 500 N–10 μm, the standard deviation decreased to ±31.1 HV_0.5_, a reduction of approximately 32.8%, demonstrating that moderate impact parameters can improve the microstructural uniformity of the surface layer. This is mainly associated with the localized plastic flow and microstructural redistribution induced by the ultrasonic impact. In contrast, the standard deviation of the 1000 N–10 μm specimen increased to ±42.2 HV_0.5_. Although the average hardness was further enhanced, the more severe localized plastic deformation under the high-load condition led to certain differences in the degree of strengthening among different regions, resulting in a slight increase in hardness fluctuation.

### 3.2. Microstructure

#### 3.2.1. Metallographic Structure

As shown in Figure 6, the initial microstructure of the 100CrMnMoSi8-4-6 bearing steel primarily consisted of tempered martensite, finely dispersed carbides, and a small amount of undissolved carbides. In the initial state, the carbides were relatively large in size, with some coarse blocky undissolved carbides unevenly distributed within the matrix. Since this steel is a high-carbon, high-alloy bearing steel, elements such as Cr, Mo, and Si promote carbide precipitation, endowing the material with a high initial hardness. The uniformly distributed fine carbides can retard localized plastic deformation and improve the load-bearing capacity of the surface layer [25,26]. In addition, the abundant dispersed carbides observed in the metallographic image can exert a favorable second-phase strengthening effect under friction and cyclic loading [28]. After ultrasonic impact treatment, a distinct plastic deformation strengthened layer was formed in the surface layer of the specimens. The thickness of the strengthened layer was approximately 39.71 μm under the 500 N condition, while it increased to 47.67 μm under the 1000 N condition, representing an increase of about 20.0%. This indicates that with an increased static load, the degree of plastic deformation induced by ultrasonic impact is further enhanced, and the impact stress waves propagate deeper into the material, thereby expanding the extent of the strengthened layer.

It can be observed from the microstructural morphology that after ultrasonic impact treatment, the tempered martensite structure in the surface layer was significantly refined. The original acicular or lath-like morphology gradually became indistinct, and the surface microstructure tended to be homogeneous and dense. Simultaneously, the number of large-sized carbides decreased markedly, and some coarse undissolved carbides underwent fragmentation and fracture. This is primarily because the high-frequency cyclic loading during UIT generates a high strain rate, and the surface material undergoes intense shear plastic deformation, causing the more brittle carbides to fracture and become refined, as well as to redistribute along the direction of plastic flow. The larger the carbide size, the more likely the interface between the carbide and the martensitic matrix becomes a preferential site for fatigue crack initiation, thereby significantly reducing the fatigue life of the material [29,30]. Through the severe plastic deformation induced by UIT, not only is the refinement and homogenization of carbides achieved but the dislocation density is also substantially increased, forming a synergistic effect of grain refinement strengthening and dispersion strengthening, which is beneficial in suppressing the early initiation and propagation of fatigue cracks.

Compared with the 500 N specimen, the microstructural refinement in the surface layer was more pronounced under the 1000 N condition, and the degree of carbide dispersion was further enhanced. This indicates that the higher impact load intensifies dislocation multiplication and grain distortion, promoting further fragmentation and homogenization of the carbides. Meanwhile, the refined carbides can exert a stronger pinning effect on dislocation motion, thereby improving the work hardening capacity and microhardness of the material. This corresponds well with the previous hardness results, in which the average hardness of the 1000 N specimen reached 803.9 ± 42.2 HV_0.5_.

However, since it is difficult to clearly resolve nano/submicron-scale microstructural evolution using conventional metallographic images at the 10 μm scale, Figure 6c alone is insufficient to quantitatively characterize the grain size and local orientation changes. Therefore, subsequent EBSD analysis was conducted to examine the surface-layer grain size, Kernel Average Misorientation (KAM), and recrystallization characteristics, in order to further reveal the grain refinement mechanism and plastic deformation behavior induced by ultrasonic impact treatment. The EBSD results can more accurately reflect the high-density dislocation accumulation, grain boundary multiplication, and the degree of grain refinement in the surface layer, thereby providing a quantitative basis for the strengthening mechanisms.

#### 3.2.2. EBSD Results

To quantitatively reveal the regulation mechanism of the ultrasonic impact static load on the gradient plastic deformation, grain refinement, dislocation accumulation, and phase distribution in the surface layer of the 100CrMnMoSi8-4-6 bearing steel, multi-dimensional EBSD characterization was carried out on the surface strengthened layers of the specimens treated under the 500 N–10 μm and 1000 N–10 μm conditions. The results are presented in Figure 7, where Figure 7a corresponds to the 500 N condition and Figure 7b to the 1000 N condition. Subfigure 1 shows the Inverse Pole Figure (IPF) map, Subfigure 2 the Kernel Average Misorientation (KAM) map, Subfigure 3 the statistical distribution of grain size, Subfigure 4 the distribution of low- and high-angle grain boundaries, Subfigure 5 the phase distribution map, and Subfigure 6 the quantitative statistics of phase fractions.

The IPF maps directly reflect the lath fragmentation, lattice rotation, and grain refinement behavior of the tempered martensite matrix under the high-frequency ultrasonic impact plastic shearing. As can be seen from Figure 7(a-1), after treatment with a static load of 500 N, the original acicular tempered martensite laths were noticeably twisted and segmented. Numerous orientation partitions appeared within the grains, with local sub-grain blocks distributed in an interlaced manner, although the elongated outlines of the original martensite could still be identified. When the static load was increased to 1000 N (Figure 7(b-1)), the original martensite lath morphology in the surface layer essentially disappeared, the color distribution of the crystal orientations became more dispersed and chaotic, and the degree of lattice rotation was significantly enhanced. This phenomenon originates from the reinforcing effect of the static load on the transmission of ultrasonic impact energy: a higher static load increases the Hertzian contact stress between the impact ball head and the specimen, enabling the high-frequency cyclic shear stress waves to propagate deeper into the material and inducing a greater cumulative equivalent plastic strain. As a result, the martensite matrix undergoes thorough shear fragmentation and lattice rearrangement, and the degree of microstructural homogenization is markedly improved. Combined with the previous metallographic observations, where the thickness of the plastic deformation strengthened layer was 39.71 μm under the 500 N condition and increased to 47.67 μm under the 1000 N condition, the extensively orientation-disturbed deformation zone in the IPF maps corresponds to this thickened plastic strengthened layer. This demonstrates that increasing the static load can simultaneously expand the depth of the grain-refined gradient layer and the degree of refinement.

Kernel Average Misorientation (KAM) is primarily used to characterize local crystallographic misorientation, orientation gradients, and lattice distortion, and can indirectly reflect local orientation changes associated with plastic deformation. Higher KAM values generally correspond to more pronounced local lattice distortion and orientation gradients and may be associated with deformation behavior related to geometrically necessary dislocations (GNDs) during plastic deformation. It should be noted that KAM is affected by several factors, including the EBSD step size, kernel definition, data quality, and post-processing procedure. Therefore, in the present study, KAM is not regarded as a direct quantitative measure of dislocation density but is mainly used for the qualitative or semi-quantitative evaluation of the degree of local plastic deformation and lattice distortion.

As shown in Figure 7(a-2,b-2), both specimens exhibit relatively high KAM values near the surface, which gradually decrease with increasing depth, consistent with the gradient plastic deformation induced by UIT. Compared with the 500 N specimen, the 1000 N specimen exhibits a larger fraction of regions with relatively high KAM values, indicating more pronounced local crystallographic misorientation, lattice distortion, and plastic deformation in the surface layer after treatment at the higher static load. Under the higher static load, the more intense plastic deformation may promote dislocation-related slip, interaction, and rearrangement, thereby producing more pronounced local orientation gradients and lattice distortion. However, these results alone cannot be used to directly quantify the actual dislocation density.

This more pronounced plastic deformation state may contribute to surface strengthening. On the one hand, dislocation-related interactions and rearrangements during plastic deformation may contribute to work hardening, which is consistent with the higher surface hardness observed for the 1000 N specimen. On the other hand, severe plastic deformation may promote the subdivision and refinement of the original martensitic laths and the formation of substructures, thereby further modifying the near-surface microstructure.

The statistical distribution of grain size is presented in Figure 7(a-3,b-3). The average grain size in the surface layer of the 500 N–10 μm specimen was 1.54 μm; after increasing the static load to 1000 N, the average surface grain size was refined to 1.44 μm. The grain refinement induced by ultrasonic impact follows the classical plastic refinement pathway: dislocation multiplication leads to the formation of dislocation cells, which subsequently results in the generation of subgrains and ultimately the transformation into high-angle grain boundaries. Under the lower static load of 500 N, the plastic strain is limited, and only some of the coarse martensite laths are subdivided into micron-sized subgrains. In contrast, the higher static load of 1000 N introduces a greater equivalent plastic strain, causing the dislocation cell walls to continuously absorb mobile dislocations. The misorientation between subgrains gradually increases, and a large number of low-angle subgrain boundaries transform into high-angle grain boundaries, eventually leading to equiaxed grain refinement. Grain refinement strengthening is a key mechanism for improving rolling contact fatigue performance. Refined grains substantially increase the total grain boundary area, and grain boundaries can effectively hinder the propagation of fatigue cracks along slip bands. Moreover, uniform and fine grains reduce local stress concentrations and mitigate the tendency for crack initiation at the interfaces between coarse carbides and the martensitic matrix. This can reasonably explain the experimental observations in the fatigue tests, where the 1000 N specimen exhibited smaller fatigue spalling pits and less severe surface damage.

The grain boundary distribution maps are shown in Figure 7(a-4,b-4), where red lines represent low-angle grain boundaries (LAGBs, i.e., subgrain boundaries) with misorientations of 2–10°, and black lines represent high-angle grain boundaries (HAGBs) with misorientations > 10°. Quantitative statistics indicate that in the 500 N specimen, the fraction of LAGBs was 9.1% and that of HAGBs was 90.9%. After increasing the static load to 1000 N, the LAGB fraction increased to 10.7%, while the HAGB fraction decreased to 89.3%. LAGBs are formed by dislocation tangles and the interweaving of slip bands, and their increased proportion directly reflects the enhanced degree of plastic deformation in the material. Under the 1000 N condition, the higher cyclic shear strain continuously multiplies dislocations, and a large number of dislocations rearrange to form subgrain boundaries, resulting in a significant increase in the LAGB fraction. These low-angle subgrain boundaries can pin dislocation motion, further enhancing the work hardening effect in the surface layer. Meanwhile, the ongoing deformation progressively increases the misorientation of some subgrains, causing them to transform into high-angle grain boundaries and thereby creating a graded composite grain boundary structure of “subgrains–fine grains”. This composite grain boundary system combines the synergistic effects of dislocation strengthening and grain refinement strengthening, continuously impeding dislocation slip under alternating rolling contact stress and retarding the early nucleation of microcracks.

The phase distribution maps (Figure 7(a-5,b-5)) and the quantitative phase fraction statistics (Figure 7(a-6,b-6)) show that the red regions correspond to the body-centered cubic (BCC) tempered martensite matrix, the green regions represent cementite (Fe_3_C) carbides, and trace impurity phases are surface oxidation products generated during the measurement process. Quantitative statistics reveal that the martensite fraction in the 500 N specimen is 87.2% and the cementite fraction is 2.9%, while in the 1000 N specimen the martensite fraction increases to 88.3% and the cementite fraction decreases to 2.2%. Under high-static-load ultrasonic impact, the volume fraction of the cementite phase decreases slightly, and the carbides become more uniformly distributed within the matrix, which is consistent with the previously observed fragmentation, refinement, and dispersion of coarse undissolved carbides in the metallographic examination. In the high-carbon bearing steel 100CrMnMoSi8-4-6, the original bulky carbides are a brittle secondary phase, and the phase interfaces between the carbides and the martensite matrix act as preferential sites for rolling contact fatigue crack initiation. The intense shear stress induced by ultrasonic impact can fracture and break the coarse carbides into submicron fine particles; the reduced carbide size and their dispersion within the matrix significantly weaken the stress concentration effect at the carbide–matrix interfaces, thereby lowering the probability of crack initiation. The total fraction of surface oxide phases is below 5%, which has no apparent detrimental effect on the rolling contact fatigue performance of the matrix.

However, the limitations of EBSD analysis should also be considered. The decrease in the EBSD-identified cementite fraction may be associated with the refinement and fragmentation of cementite induced by the severe plastic deformation during UIT. As the carbide size decreases and the degree of deformation and crystal defects increases, some fragmented fine carbides may no longer provide diffraction patterns of sufficient quality for reliable EBSD phase indexing, resulting in an apparent reduction in the identifiable cementite fraction. Therefore, the EBSD phase-fraction results should be interpreted in conjunction with the observed changes in carbide morphology and should not be regarded independently as direct evidence of cementite dissolution or an actual reduction in the cementite volume fraction.

Based on the multidimensional EBSD characterization results, the effects of UIT static load on the near-surface microstructure of 100CrMnMoSi8-4-6 bearing steel and its possible relationship with rolling contact fatigue behavior can be further discussed. As the UIT static load increased from 500 N to 1000 N, the near-surface region exhibited more pronounced grain refinement, local lattice distortion, carbide fragmentation, and changes in grain-boundary characteristics. Together with the increases in hardness and compressive residual stress, these microstructural changes indicate that the higher static load produced a more pronounced surface-strengthened state. The resulting grain refinement, plastic-deformation-related strengthening, carbide refinement, and grain-boundary evolution may collectively contribute to the improvement in rolling contact fatigue performance.

Under the 1000 N condition, the martensitic microstructure exhibited more pronounced fragmentation and refinement, with the average near-surface grain size decreasing to 1.44 μm. The increased number of grain boundaries may contribute to improving the resistance of the material to localized plastic deformation and fatigue crack propagation. Meanwhile, the 1000 N specimen exhibited higher KAM values, indicating more pronounced local crystallographic misorientation, orientation gradients, and lattice distortion in the near-surface region, which may be associated with more intense plastic deformation and dislocation-related activity. In addition, the fragmentation and refinement of coarse carbides observed after UIT may help reduce local microstructural heterogeneity and may decrease the tendency for microcrack initiation near the carbide–matrix interfaces. Changes in the characteristics of low-angle and high-angle grain boundaries may also influence the mechanical response of the strengthened layer. Overall, these microstructural changes exhibit trends consistent with the increases in hardness and compressive residual stress and the improvement in rolling contact fatigue performance observed after UIT. Under the experimental conditions investigated in the present study, the 1000 N–10 μm specimen exhibited the highest rolling contact fatigue life, whereas the improvement in fatigue life under the 500 N condition was comparatively smaller.

## 4. Discussion

### 4.1. Spalling Pit Morphology

As shown in Figure 8, the surface damage morphologies of the 100CrMnMoSi8-4-6 bearing steel after rolling contact fatigue testing exhibited significant differences depending on the ultrasonic impact treatment parameters. The surface of the 500 N–10 μm specimen displayed a large number of fine spalling pits accompanied by localized larger fatigue spalling areas, whereas the surface of the 1000 N–10 μm specimen remained more intact overall, with only a few isolated spalling pits observed and a markedly reduced degree of surface damage. This indicates that the strengthened layer formed under a higher impact load can effectively enhance the material’s resistance to rolling contact fatigue damage.

As can be seen from Figure 8(a-2,a-3), the 500 N specimen exhibited a large number of densely distributed fine spalling pits within the contact region. Meanwhile, in some areas, the spalling pits expanded and coalesced, forming a large-area fatigue spalling zone with a length of approximately 150.28 μm and a width of 36.17 μm. This indicates that significant accumulated fatigue damage had already occurred in the surface layer under the action of cyclic contact stress. Due to the relatively limited thickness of the strengthened layer and the degree of surface strengthening under the 500 N condition, the cyclic shear stress more readily induced microcrack initiation in the near-surface region. After the cracks propagated along the direction of maximum shear stress, they eventually led to the detachment of surface material and the formation of dense pitting pits. Under continued cyclic loading, these eventually evolved into larger spalling pits.

In contrast, the 1000 N–10 μm specimen exhibited only a few localized spalling pits after the rolling contact fatigue test, and the size of the spalled regions was significantly reduced. This indicates that the strengthened layer formed under a higher impact load can more effectively inhibit the initiation and propagation of fatigue cracks. On one hand, the higher surface hardness (803.9 ± 42.2 HV_0.5_) achieved under the 1000 N condition enhanced the material’s resistance to plastic deformation; on the other hand, the combined effect of a deeper plastically strengthened layer and a higher level of residual compressive stress effectively reduced the accumulation of cyclic contact stress-induced damage in the subsurface region, thereby delaying the progression of fatigue spalling.

As shown in Figure 8(d-3), for the 1000 N specimen, the surface roughness further decreased to 0.09 μm, representing a reduction of approximately 18%. After the rolling contact fatigue test, the surface profile undulations were significantly diminished, and the curve fluctuations became more gentle, indicating that the surface micro-topography was further “leveled” during the rolling contact process. This phenomenon suggests that, under certain contact load and lubrication conditions, rolling contact does not necessarily lead to surface roughening; on the contrary, it may produce a certain “surface self-leveling effect.” During the cyclic rolling process, the contact zone is subjected to continuous normal pressure and tangential shear stress, inducing micro-plastic flow of the surface material, which gradually flattens the original turning marks. Meanwhile, the local asperity peaks undergo plastic extrusion and material transfer under repeated contact, with some material flowing into the low-lying areas and thereby producing a “filling” effect on the micro-pits. Consequently, shallow but continuous machining textures could still be observed on the 1000 N specimen, while the overall roughness was significantly reduced. At the same time, the high-hardness strengthened layer formed by ultrasonic impact treatment enhanced the surface resistance to spalling, allowing for the contact stress to be distributed more uniformly over the surface and reducing the local stress concentration effect. In particular, under the 1000 N condition, the thickness of the strengthened layer reached approximately 47.67 μm, an increase of about 20% compared with the 39.71 μm of the 500 N specimen, which could more effectively buffer the alternating shear stress generated during rolling contact. As a result, only a few isolated spalling pits formed on its surface, without the occurrence of large-area continuous spalling.

### 4.2. Work Hardening

As shown in Figure 9, the surface hardness of all specimens was further improved after rolling contact fatigue (RCF) testing. As can be seen from Figure 8a, the hardness increase after RCF was mainly concentrated in the near-surface region, with the most pronounced increment occurring within the 0–300 μm range. For the initial specimen, the average hardness before RCF (Un-RCF) was 746.12 HV_0.5_, which increased to 783.25 HV_0.5_ after RCF, an increment of approximately 4.98%. For the 500 N–10 μm specimen, the hardness increased from 789.93 HV_0.5_ to 818.62 HV_0.5_, an increment of about 3.63%; for the 1000 N–10 μm specimen, the hardness was further enhanced from 811.72 HV_0.5_ to 828.81 HV_0.5_, an increment of about 2.11%. This indicates that the periodic contact stress and alternating shear stress generated during the rolling contact process can continuously induce secondary plastic deformation in the surface layer, thereby producing a pronounced work hardening effect.

As shown in the schematic diagram of Figure 10, the “surface self-leveling effect” induced by rolling contact acts synergistically with work hardening. Under the combined action of normal contact pressure and tangential shear force, the protruding areas on the surface are continuously flattened, the original turning marks are gradually diminished, and localized material flows into the low-lying regions, producing a certain “filling” effect on micro-defects and shallow pits, thereby smoothing the surface profile. Meanwhile, the load-bearing capacity of the material is further improved after work hardening of the surface layer, which effectively reduces the degree of stress concentration in the contact region and consequently alleviates the tendency for microcrack initiation. This indicates that when an appropriate equilibrium is reached between the degree of surface strengthening and the contact stress, the rolling contact process can promote the progressive stabilization of the surface microstructure, thereby creating a “dynamic self-smoothing” state that is favorable for fatigue service.

However, as the cyclic loading continues to increase, the contact-induced work hardening may also have certain adverse effects. With the continuous accumulation of dislocations, the plastic stored energy in localized regions progressively increases, which may lead to an enhanced tendency for material embrittlement. Meanwhile, the property gradient difference between the work-hardened layer and the substrate can cause a redistribution of local shear stress. When the local stress exceeds the load-bearing limit of the strengthened layer, cracks will still preferentially initiate and propagate at turning marks, the edges of spalling pits, or microstructural defects, a phenomenon that is particularly evident in Figure 8(b-3). Therefore, moderate and stable work hardening is beneficial for improving rolling contact fatigue resistance, whereas excessive localized hardening may accelerate fatigue spalling failure in the later stages.

From the perspective of microstructural evolution and mechanical response, the secondary work hardening after rolling contact exhibited a clear dependence on the initial strengthening state. The additional hardening increments after rolling contact were 4.98%, 3.63%, and 2.11% for the initial, 500 N, and 1000 N specimens, respectively. The initial specimen, with the lowest degree of pre-strengthening, exhibited the largest subsequent hardening increment under cyclic rolling contact. After 500 N UIT, both the initial hardness and compressive residual stress were increased, while a further hardening increment of 3.63% was still observed after rolling contact, indicating that appreciable contact-induced plastic accommodation and work hardening could still occur in the surface layer. In comparison, the 1000 N treatment produced higher initial hardness and compressive residual stress, together with more pronounced grain refinement, an increased fraction of low-angle grain boundaries, and enhanced local crystallographic misorientation; however, its additional hardening increment after rolling contact decreased to 2.11%. These results indicate that the initial strengthening state established by UIT significantly influences the subsequent secondary hardening response during rolling contact.

This behavior may be associated with the degree of plastic deformation introduced during UIT and the resulting initial strengthening state. With increasing static load, the surface layer undergoes more pronounced plastic deformation before rolling contact, resulting in higher hardness, greater compressive residual stress, and more refined microstructural features. Consequently, under subsequent cyclic Hertzian contact loading, the capacity for further plastic accommodation, microstructural evolution, and work hardening may become relatively limited, resulting in a smaller secondary hardening increment.

Therefore, within the 500–1000 N static-load range investigated in this study, the material exhibited a clear static-load-dependent strengthening–hardening response. At 500 N, appreciable contact-induced hardening was retained after the establishment of the initial surface strengthening state. At 1000 N, a stronger initial strengthening state was established and, although the secondary hardening increment was smaller, the highest rolling contact fatigue life was obtained. This indicates that the magnitude of secondary hardening alone does not determine the final fatigue performance; rather, the UIT-induced hardness, compressive residual stress, and microstructural state should be considered together with the subsequent cyclic contact response. Under the present experimental conditions, the 500 N condition exhibited a relatively balanced combination of initial strengthening and subsequent hardening capacity, whereas the 1000 N condition provided stronger initial strengthening and the highest fatigue life. Since only two UIT static loads, 500 N and 1000 N, were investigated, the present results are more appropriately interpreted as evidence of a static-load-dependent strengthening–hardening response, rather than as establishing a universal critical static load or optimal processing range. Further validation over a broader static-load range, with different materials and rolling contact conditions, is required to determine the general applicability of this relationship.

The above results indicate a clear static-load-dependent response during ultrasonic impact treatment. The 500 N and 1000 N conditions produced different initial surface-strengthening states and subsequent rolling-contact-induced hardening responses. At 500 N, the pre-strengthening effect improved the resistance of the surface layer to initial plastic deformation while retaining a relatively pronounced capacity for subsequent contact-induced hardening. In comparison, although the 1000 N condition produced a stronger initial strengthening effect, the contribution from further hardening during subsequent rolling contact was relatively limited. These observations suggest that the balance between the initial strengthening state of the surface layer and its capacity for further plastic hardening is strongly influenced by the applied static load. Under the present experimental conditions, the 500 N treatment exhibited a more favorable balance between initial strengthening and subsequent hardening capacity, resulting in a relatively pronounced interaction between initial static strengthening and subsequent dynamic contact hardening. It should be emphasized, however, that only two static loads, 500 N and 1000 N, were investigated in the present study; therefore, the available results are insufficient to establish a strict critical static-load threshold or an optimal load range. Future studies covering a broader and more closely spaced range of static loads are required to further identify the transition characteristics and determine the optimal processing window. Nevertheless, the observed interaction between static-load-dependent pre-strengthening and subsequent contact-induced hardening provides further insight into the dynamic service response of UIT-treated surfaces and may serve as a useful reference for optimizing the parameters of an integrated surface finishing and strengthening process for wind-turbine bearing rollers.

### 4.3. Fatigue Life

In this study, rolling contact fatigue tests were carried out on the initial specimen, the 500 N–10 μm UIT specimen, and the 1000 N–10 μm UIT specimen. The fatigue life results of the different specimens are presented in Figure 11. As can be seen from the figure, ultrasonic impact treatment can significantly improve the rolling contact fatigue life of 100CrMnMoSi8-4-6 bearing steel. The contact fatigue life of the initial specimen was 0.47 × 10^7^ cycles. After treatment with 500 N–10 μm UIT, the fatigue life increased to 2.25 × 10^7^ cycles, an increase of approximately 378% compared with the initial specimen, and the life reached 4.79 times that of the original specimen. When the ultrasonic impact load was further increased to 1000 N, the contact fatigue life of the specimen reached 6.18 × 10^7^ cycles, which is approximately 1214% higher than that of the initial specimen, corresponding to 13.15 times the life of the original specimen.

The substantial improvement in fatigue life is primarily attributed to the comprehensive strengthening effect of ultrasonic impact treatment on the surface microstructure and mechanical properties of the material. UIT significantly increased the surface hardness, from 750.7 ± 46.3 HV_0.5_ for the initial specimen to 803.9 ± 42.2 HV_0.5_ for the 1000 N–10 μm specimen, thereby enhancing the surface layer’s resistance to cyclic plastic deformation. Furthermore, UIT formed plastically strengthened layers with thicknesses of approximately 39.71 μm and 47.67 μm under the 500 N and 1000 N conditions, respectively, and introduced high-level residual compressive stresses, which effectively counteracted the tensile stress components generated during rolling contact, thus delaying fatigue crack initiation. The grain refinement and the homogenized dispersion of carbides induced by UIT further improved the strength and toughness of the surface layer, providing a microstructural foundation for the enhancement of fatigue life.

Combined with the surface morphology analysis after contact fatigue, it can be observed that the surface of the initial specimen and the specimen with a lower degree of strengthening were more prone to the formation of dense spalling pits. Surface defects such as turning marks gradually evolved into crack initiation sources and eventually developed into large-area fatigue spalling. In contrast, the surface of the 1000 N–10 μm specimen exhibited only a few isolated spalling pits, and its surface integrity was significantly superior to that of the other specimens. Meanwhile, the surface self-leveling effect generated during the rolling contact process gradually flattened the original machining textures, further reducing the surface roughness; the contact-induced work hardening continuously increased the surface hardness. Together, these effects promoted the formation of a stable state characterized by “low roughness–high hardness–low stress concentration” at the contact interface, thereby significantly inhibiting crack initiation and propagation.

UIT builds a strengthened layer with high hardness, high residual compressive stress, and gradient strengthening characteristics in the material surface by promoting the refinement of tempered martensite, the fragmentation and dispersion of carbides, dislocation multiplication, subgrain formation, and grain refinement. During the rolling contact process, this strengthened layer further synergizes with contact-induced work hardening and the surface self-leveling effect to significantly suppress fatigue crack initiation and propagation, ultimately enabling the 1000 N–10 μm specimen to achieve a fatigue life of 6.18 × 10^7^ cycles, exhibiting the most superior rolling contact fatigue performance. To better characterize the distribution of fatigue life, a two-parameter Weibull distribution analysis was employed; the expression is shown in Equation (2). The shape parameter *β* and characteristic life parameter *η* were estimated using the maximum likelihood estimation (MLE) method, the relevant data is shown in Table 2, with the location parameter fixed at zero. Considering that only three independent specimens were tested for each condition, the Weibull analysis in this study was primarily used to describe the fatigue-life distribution characteristics of the present experimental dataset, rather than to establish statistically significant differences in fatigue-life distributions among the different treatment conditions.

(2) $$F(N)=1-e^{{\left(-\frac{N}{\eta }\right)}^{\beta }}$$

Here, *β* is the shape parameter, and *η* is the characteristic life parameter.

The two-parameter Weibull fitting results showed that the shape parameters *β*, of the initial, 500 N, and 1000 N specimens, were 4.34, 3.30, and 7.26, respectively. The relatively larger shape parameter obtained for the 1000 N group indicates a more concentrated fatigue-life distribution within the present dataset, whereas the 500 N group exhibited greater fatigue-life scatter. Meanwhile, the characteristic life, *η*, increased from 0.518 × 10^7^ cycles for the initial condition to 2.556 × 10^7^ and 6.584 × 10^7^ cycles for the 500 N and 1000 N conditions, respectively, indicating a substantial improvement in fatigue-life level after UIT. It should be emphasized that the present fatigue results are based on only three specimens for each condition and are limited to 100CrMnMoSi8-4-6 bearing steel under the UIT parameters and laboratory rolling contact conditions investigated in this study. Therefore, these results demonstrate the experimental trends observed under the present conditions rather than providing a general validation for complete wind-turbine bearing systems. Further studies with larger sample sizes and more representative bearing contact configurations are required to establish statistically robust fatigue-life distributions and broader engineering applicability.

The present results demonstrate that, under the investigated experimental conditions, UIT can modify the rolling contact fatigue response of 100CrMnMoSi8-4-6 roller material by reducing surface roughness, increasing surface-layer hardness, and introducing a gradient compressive residual-stress field. However, the applicability boundaries of these results should be recognized. Rolling elements, inner rings, and outer rings in actual bearings have different kinematic relationships, and local material points in these components therefore experience different contact frequencies, cyclic stress histories, and cumulative loading histories. In addition, differences in hardness, heat-treatment condition, and microstructure may exist between rolling elements and raceways, all of which can influence local cyclic plasticity, fatigue-crack initiation, and rolling contact fatigue life. In particular, for raceways with different material states and cyclic loading histories, the interaction between the UIT-induced strengthened layer and the subsurface Hertzian stress field may differ from that observed in the rollers investigated here.

Therefore, the present study demonstrates the potential of UIT for the integrated surface finishing and strengthening of 100CrMnMoSi8-4-6 bearing rollers, rather than providing a general validation of fatigue-life improvement for the entire wind-turbine bearing system. Future work should extend UIT and rolling contact fatigue testing to actual inner- and outer-raceway materials under their corresponding contact conditions and consider the component-specific cyclic loading histories to evaluate the transferability of the strengthening effects observed in the present study.

## 5. Conclusions

(1) UIT simultaneously improved the surface finish and surface-layer strengthening of oilstone-finished 100CrMnMoSi8-4-6 bearing rollers. The surface roughness Ra decreased from 0.41 μm to 0.13 and 0.11 μm after 500 N and 1000 N UIT, respectively. At 1000 N, the maximum hardness approached 900 HV_0.5_, and the compressive residual stress reached approximately −842 MPa. These results demonstrate that UIT can further improve an oilstone-finished surface while introducing beneficial surface strengthening, showing potential as an integrated surface finishing and strengthening process for bearing rollers.
(2) Increasing the UIT static load produced a more pronounced initial microstructural strengthening state. From 500 N to 1000 N, the average grain size decreased from 1.54 to 1.44 μm, while the fraction of low-angle grain boundaries increased from 9.1% to 10.7%. The EBSD-identified cementite fraction decreased from 2.9% to 2.2%; however, this apparent decrease should not be directly interpreted as cementite dissolution because severe deformation and carbide refinement may affect EBSD phase indexing.
(3) The rolling contact response showed a clear dependence on the initial strengthening state. The secondary hardening increments were 4.98%, 3.63%, and 2.11% for the initial, 500 N, and 1000 N specimens, respectively, while the Weibull characteristic fatigue life increased from 0.47 × 10^7^ to 2.556 × 10^7^ and 6.184 × 10^7^ cycles. Thus, fatigue performance depends on the combined effects of initial strengthening and subsequent cyclic response rather than secondary hardening alone. Given the limited static-load levels and fatigue sample size, further studies are required to determine an optimal processing window and validate applicability to complete bearing systems.

Under rolling contact, the surface layer exhibits secondary work hardening, and its magnitude is dependent on the initial strengthening state induced by UIT. The untreated, 500 N, and 1000 N specimens exhibited hardness increases of 4.98%, 3.63%, and 2.11%, respectively. Although the 1000 N treatment produced higher initial hardness and residual compressive stress, its subsequent hardening increment was comparatively smaller, whereas the 500 N treatment showed a relatively favorable balance between initial strengthening and subsequent contact-induced hardening. These results indicate that a higher static load does not necessarily produce a greater secondary hardening response. Within the investigated conditions, the observed behavior highlights the importance of balancing the initial strengthening effect with the remaining capacity for contact-induced hardening. A definitive critical or optimum static-load range, however, requires further investigation over a broader parameter range.

## Figures and Tables

**Figure 1:**
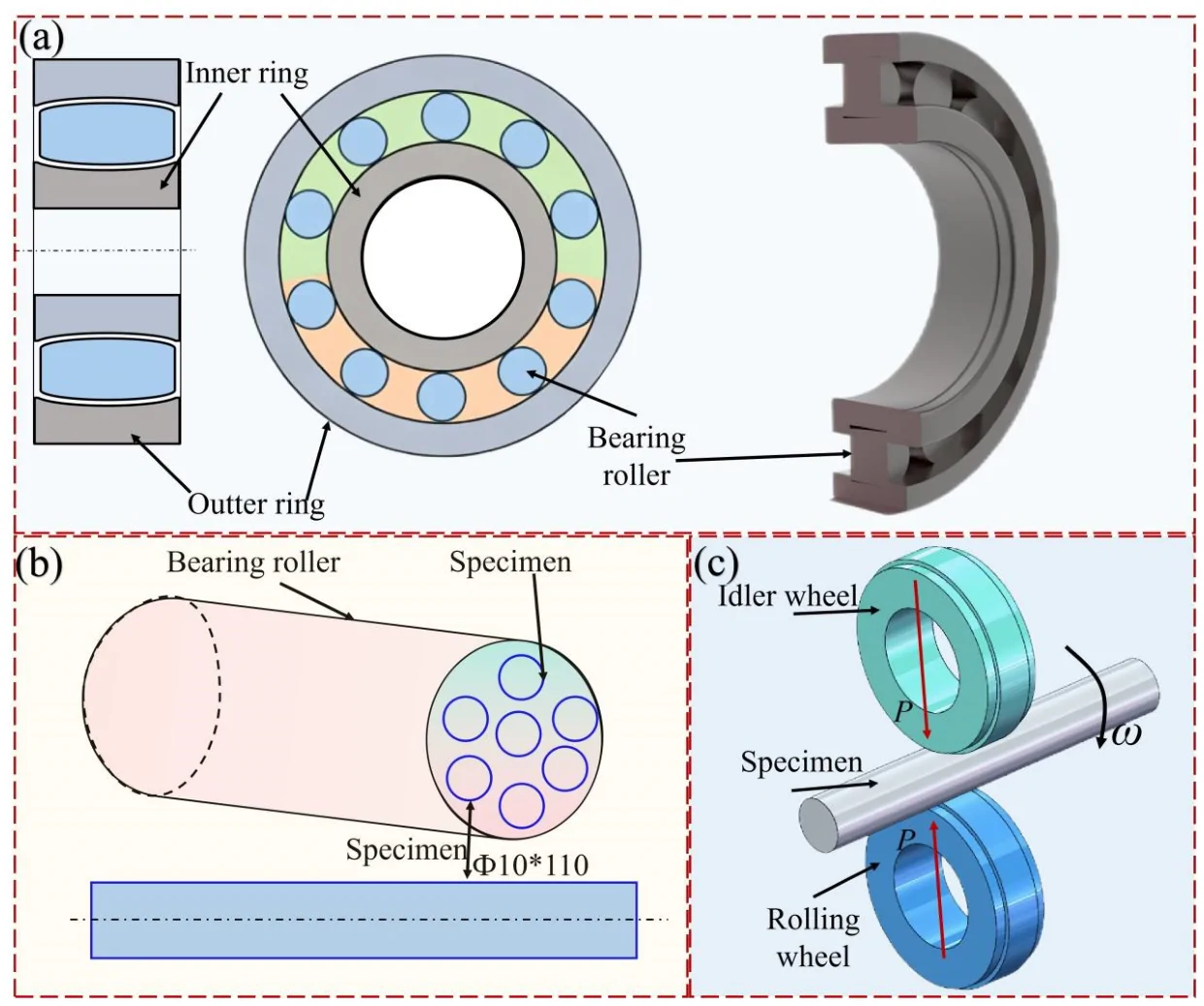
Material Sampling and Schematic Diagram. (**a**) bearing roller specimen; (**b**) specimen dimensions; (**c**) schematic diagram of the specimen contact fatigue test.

**Figure 2:**
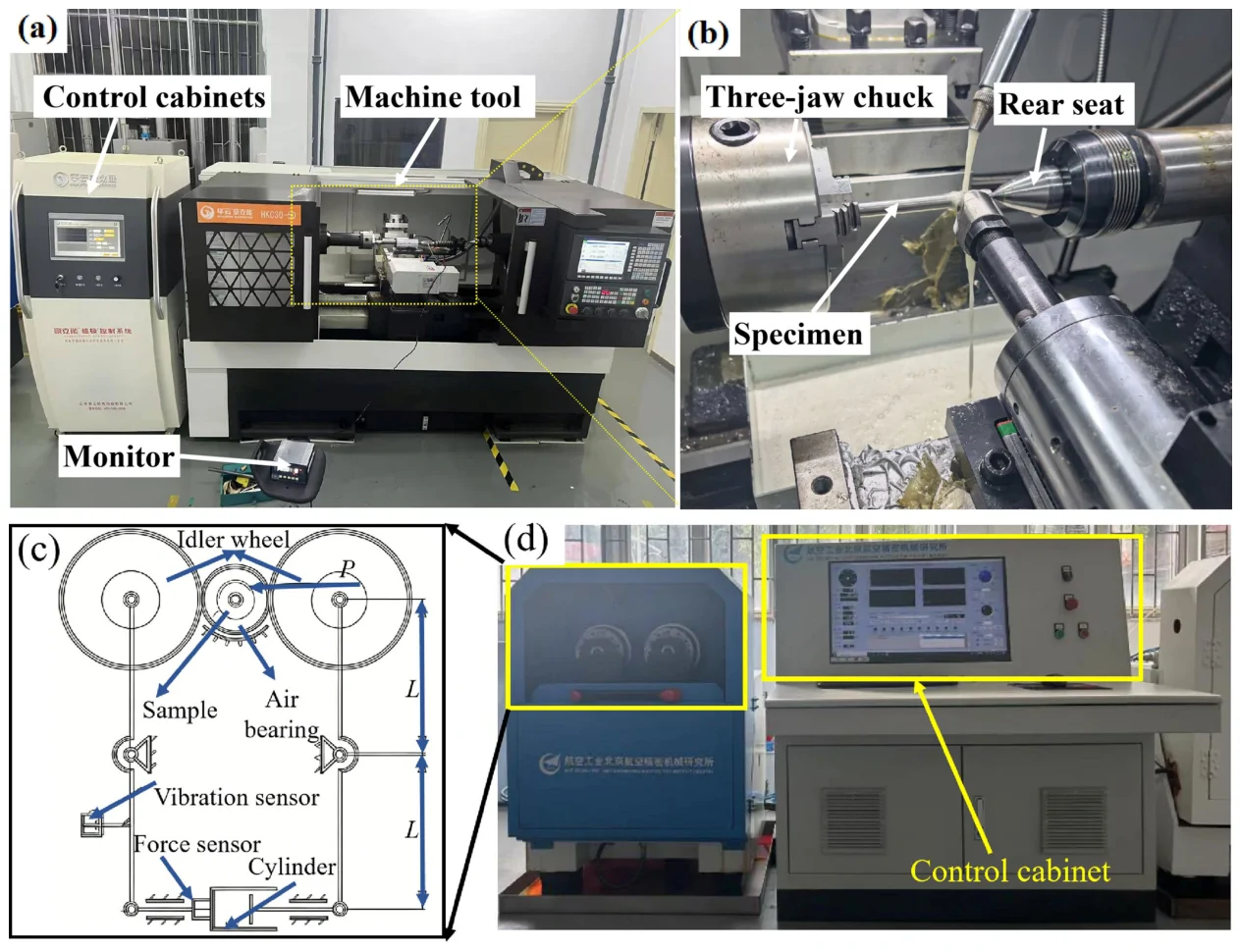
Ultrasonic impact lathe and contact fatigue testing machine. (**a**) Ultrasonic impact lathe. (**b**) Actual machining photograph. (**c**) Schematic diagram of the contact fatigue testing machine. (**d**) Contact fatigue testing machine.

**Figure 3:**
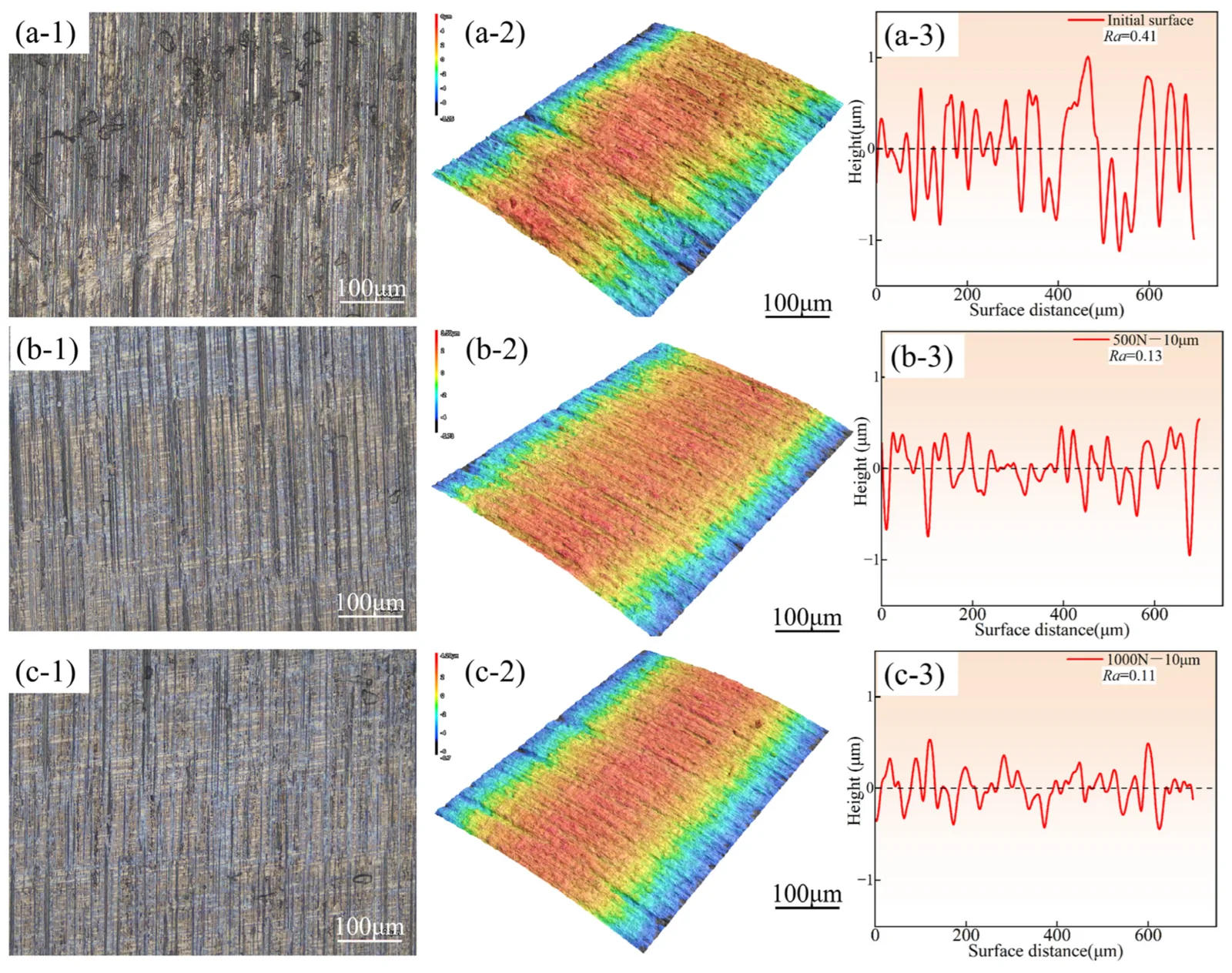
Comparison of surface roughness: (**a-1**) initial specimen surface morphology, (**b-1**) 500 N surface morphology, (**c-1**) 1000 N surface morphology, (**a-2**) initial specimen 3D topography, (**b-2**) 500 N 3D topography, (**c-2**) 1000 N 3D topography; (**a-3**) initial specimen roughness, (**b-3**) 500 N roughness, (**c-3**) 1000 N roughness.

**Figure 4:**
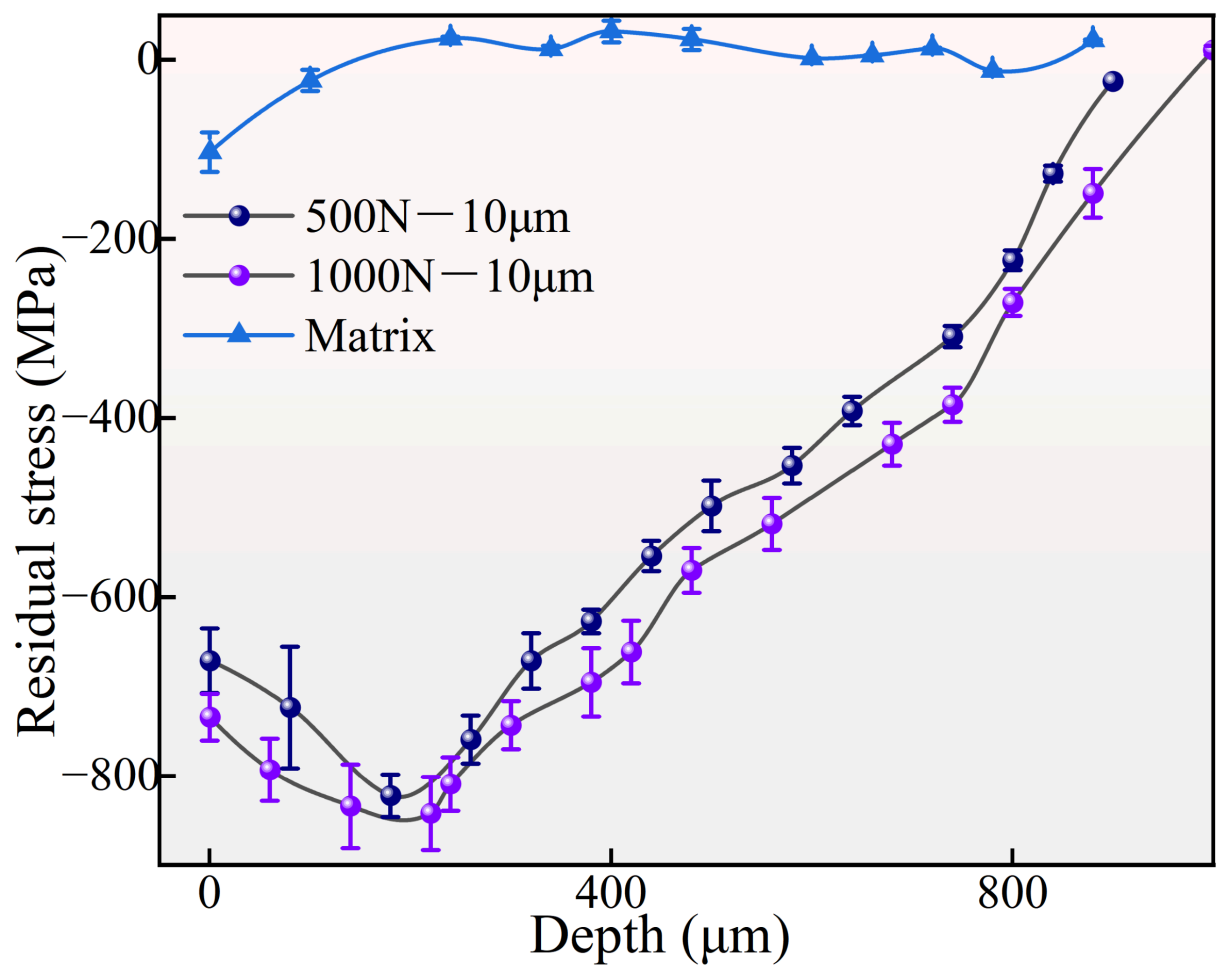
Comparison of residual stress profiles.

**Figure 5:**
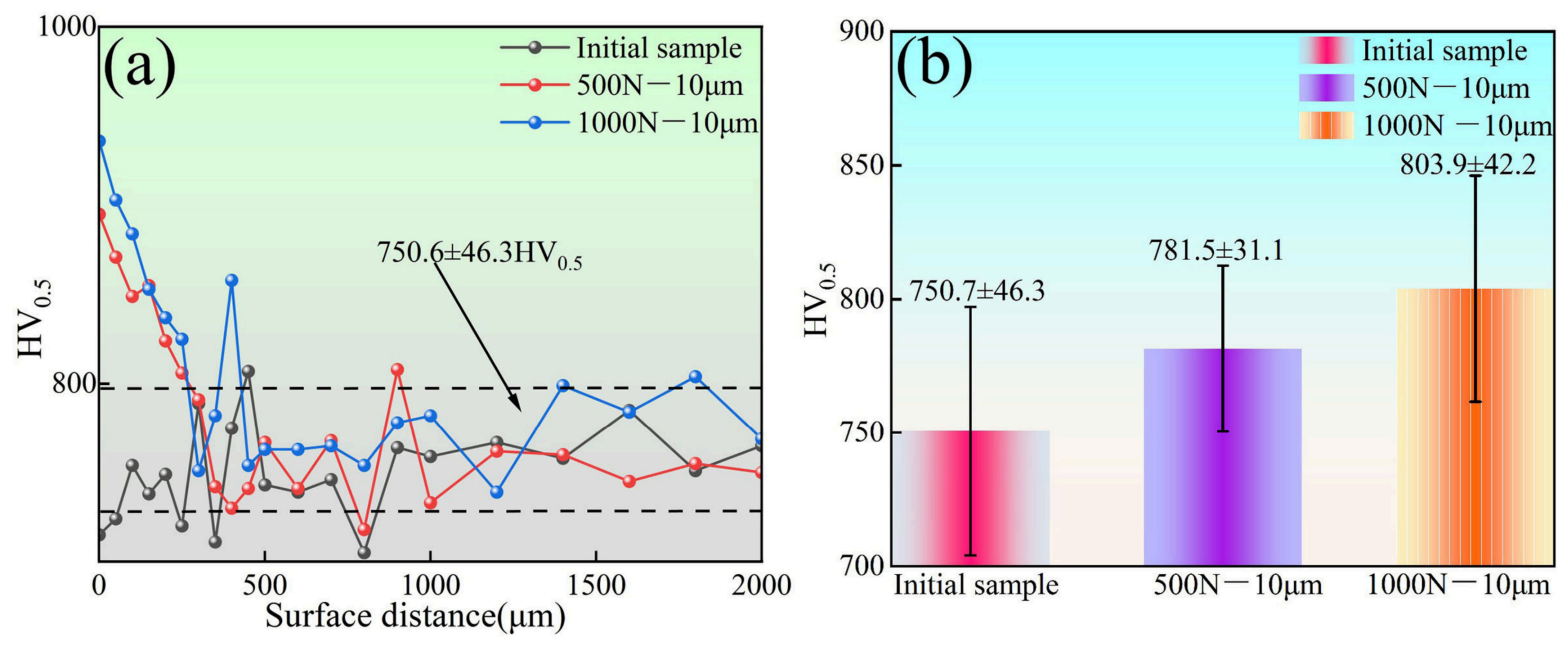
Comparison of hardness: (**a**) hardness gradient, (**b**) average hardness.

**Figure 6:**
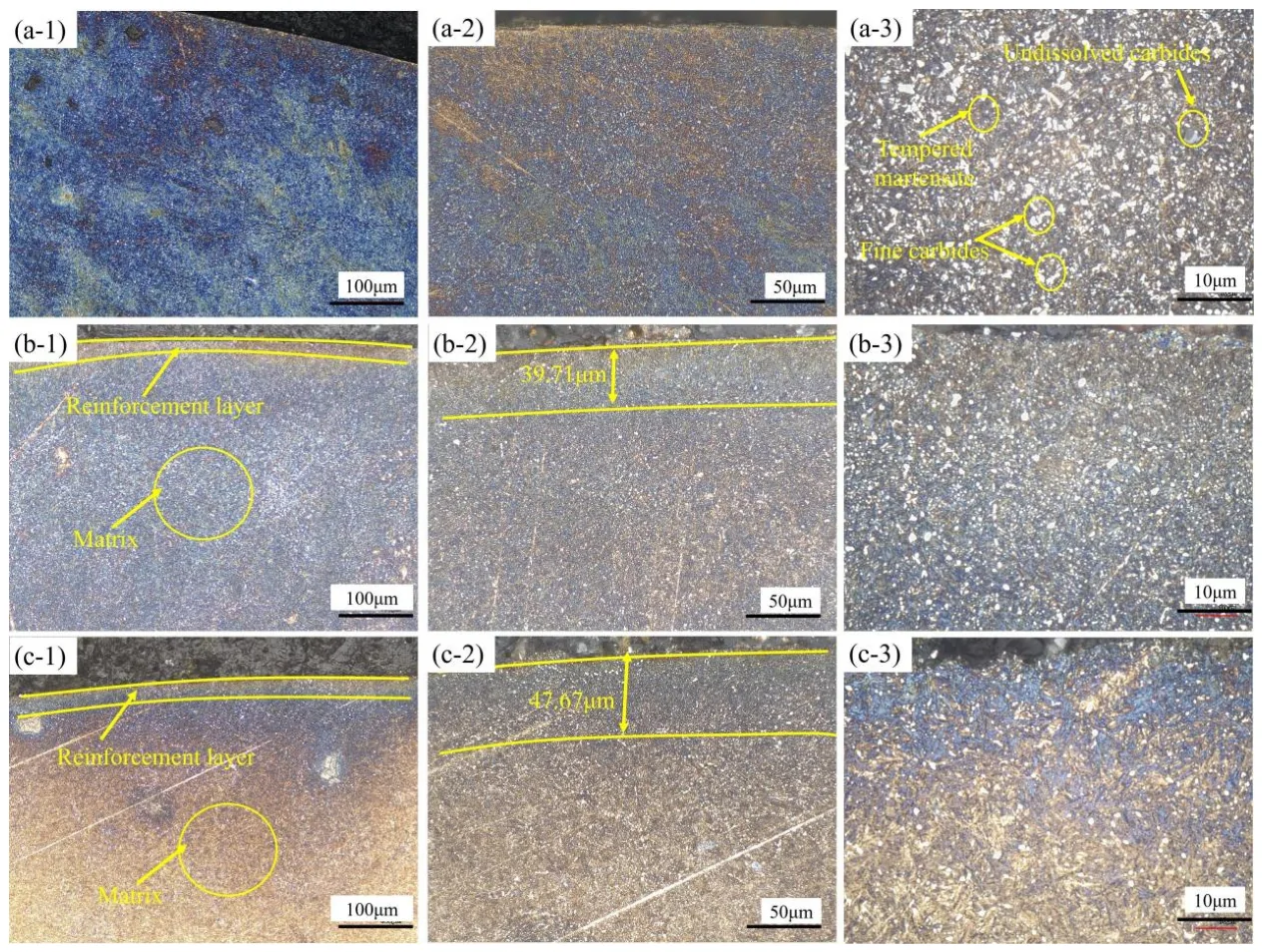
Metallographic structure results: (**a-1**) initial specimen under a 10× microscope, (**b-1**) 500 N under a 10× microscope, (**c-1**) 1000 N under a 10× microscope, (**a-2**) initial specimen under a 20× microscope, (**b-2**) 500 N under a 20× microscope, (**c-2**) 1000 N under a 20× microscope, (**a-3**) initial specimen under a 50× microscope, (**b-3**) 500 N under a 50× microscope, (**c-3**) 1000 N under a 50× microscope.

**Figure 7:**
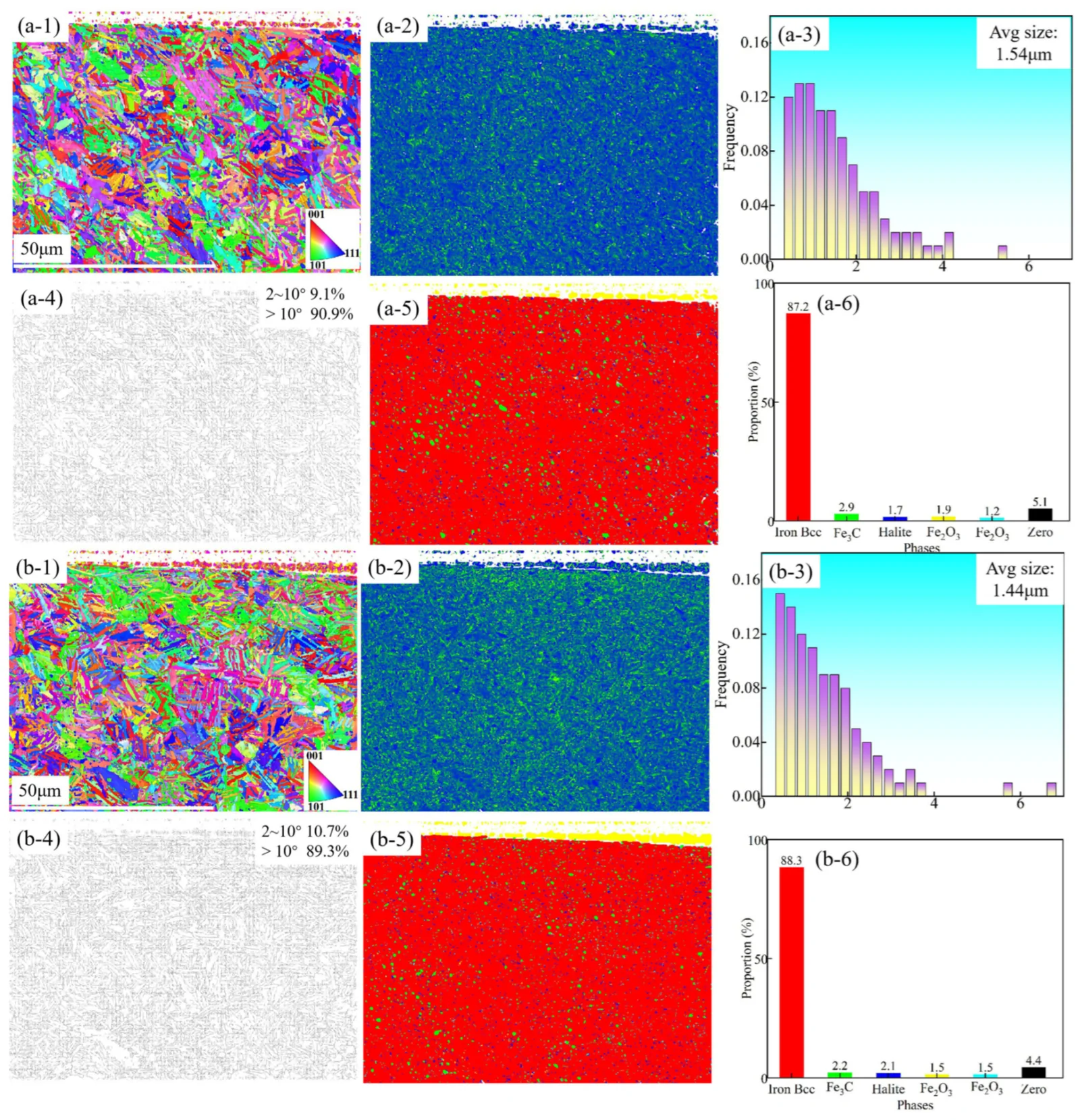
EBSD results: (**a-1**) 500 N IPF; (**a-2**) 500 N KAM; (**a-3**) 500 N Grain size; (**a-4**) 500 N Grain boundary distribution; (**a-5**) 500 N Phase distribution map; (**a-6**) 500 N Phase distribution data. (**b-1**) 1000 N IPF; (**b-2**) 1000 N KAM; (**b-3**) 1000 N Grain size; (**b-4**) 1000 N Grain boundary distribution; (**b-5**) 1000 N Phase distribution map; (**b-6**) 1000 N Phase distribution data.

**Figure 8:**
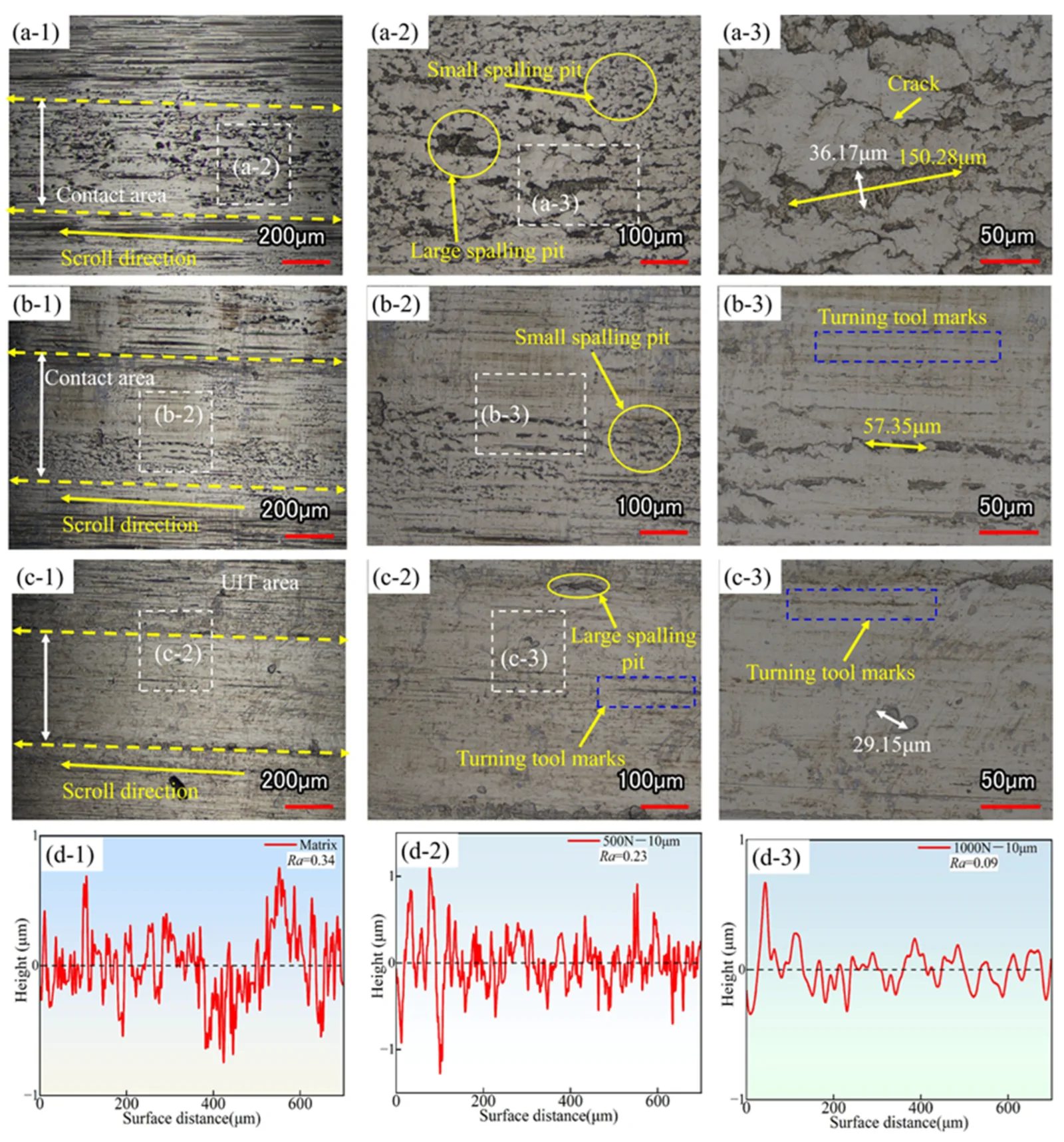
Spalling pit morphology and surface roughness:(**a-1**) Initial specimen under a 10× microscope, (**b-1**) 500 N under a 10× microscope, (**c-1**) 1000 N under a 10× microscope, (**d-1**) Initial specimen roughness. (**a-2**) Initial specimen under a 20× micro-scope, (**b-2**) 500 N under a 20× microscope, (**c-2**) 1000 N under a 20× microscope, (**d-2**) 500 N rough-ness. (**a-3**) Initial specimen under a 50× microscope, (**b-3**) 500 N under a 50× microscope, (**c-3**) 1000 N under a 50× microscope, (**d-3**) 1000 N roughness.

**Figure 9:**
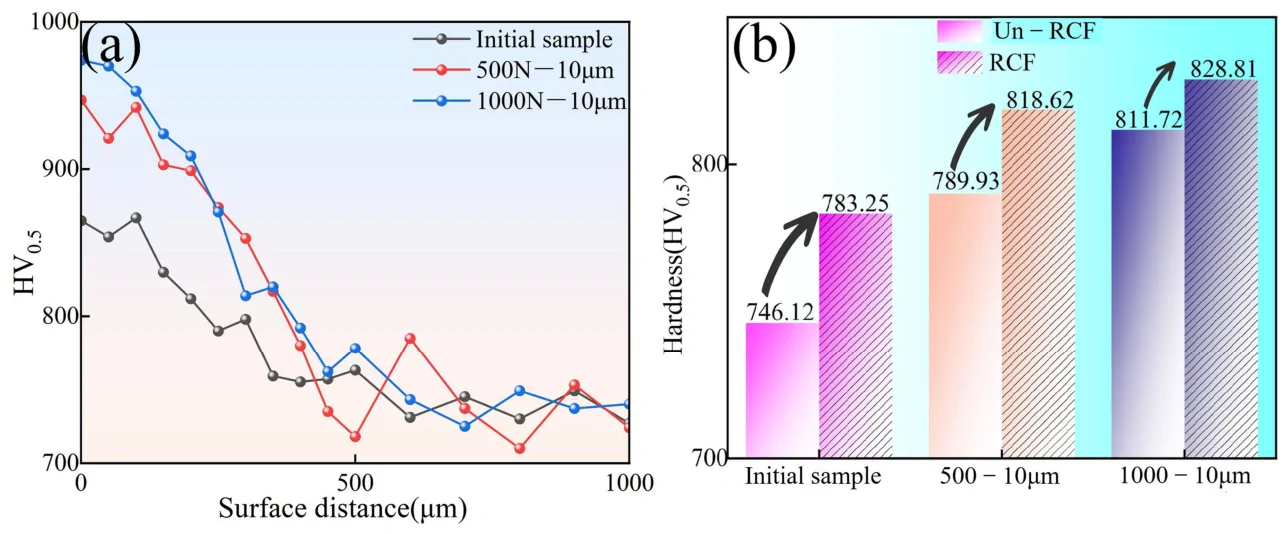
Work hardening effect: (**a**) hardness gradient, (**b**) average hardness.

**Figure 10:**
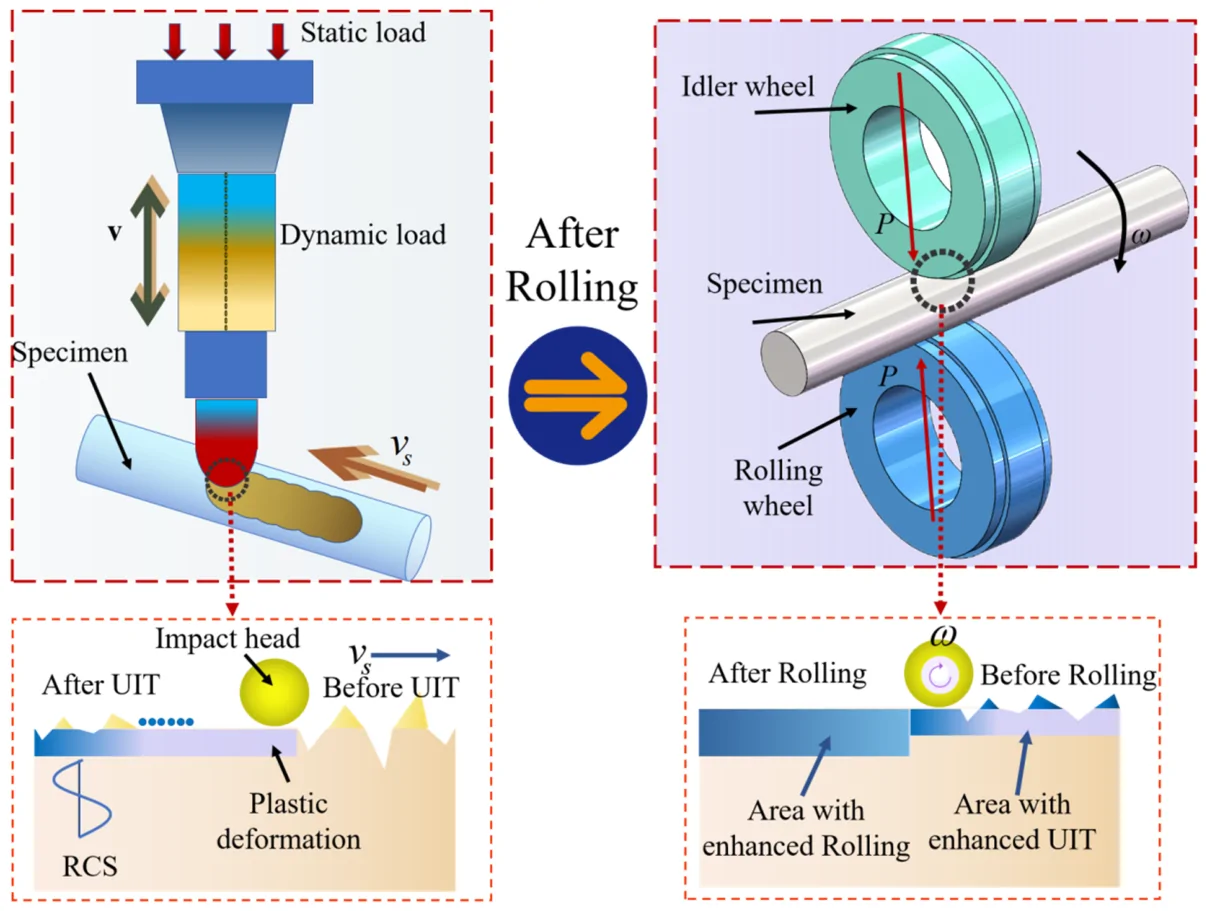
Schematic diagram of secondary hardening.

**Figure 11:**
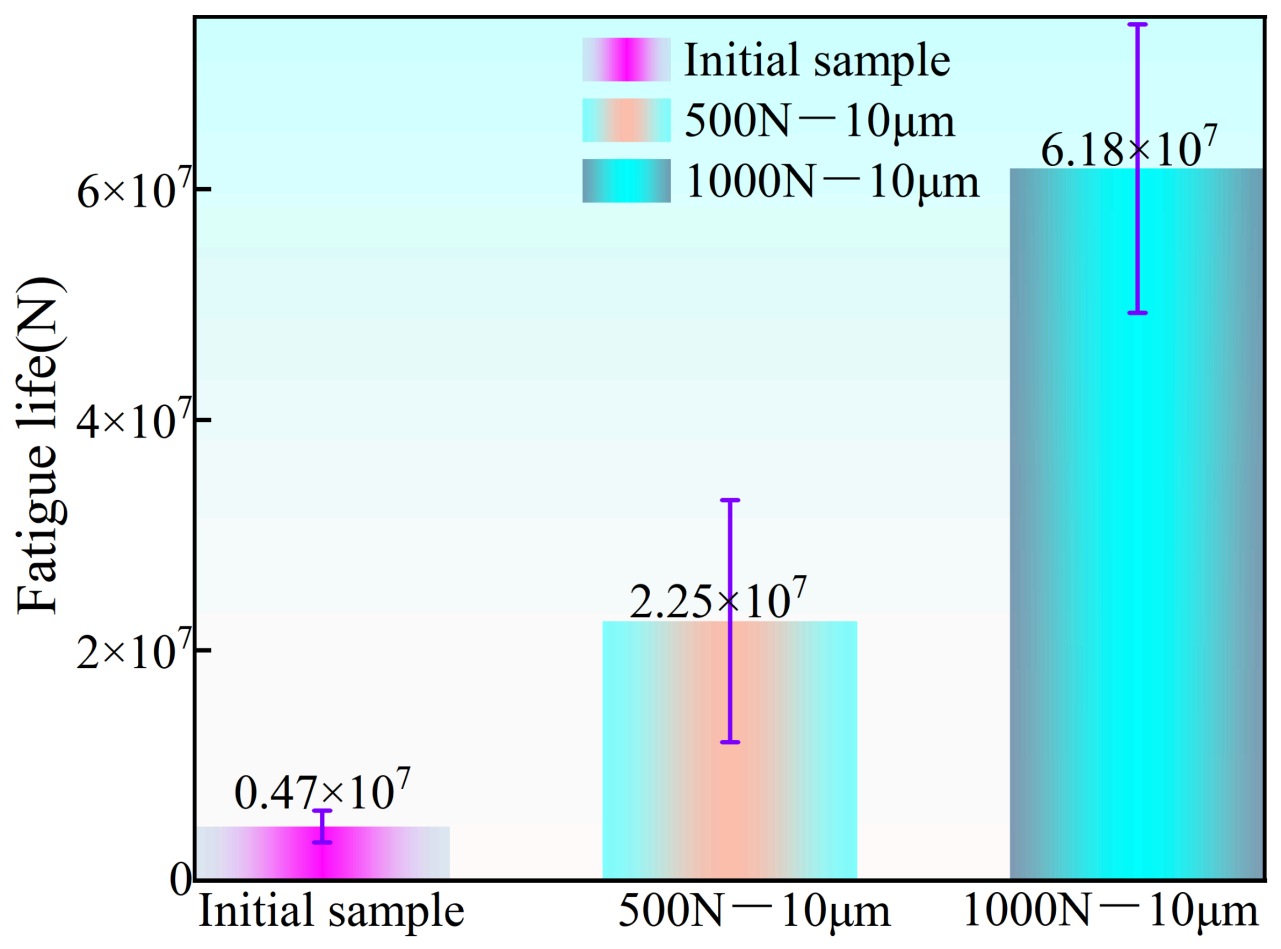
Fatigue life results.

**Table 1:** Experimental parameters.

Group	Static Load (N)	Amplitude (μm)	Preload (GPa)	Rotational Speed (r/min)
A	500	10	2.4	10,000
B	1000	10	2.4	10,000

**Table 2:** Experimental parameters.

Group	Cycle (E7 N)	*β*	*η* (×10^7^)
Initial	0.31 0.48 0.62	4.34	0.518
A	3.2 2.1 1.44	3.43	2.509
B	7.43 5.37 5.74	7.26	6.584

## Data Availability

The original contributions presented in the study are included in the article; further inquiries can be directed to the corresponding author.
